# Molecularly Imprinted Electrochemical Sensors for Halogenated anti-Infective Agent Detection: A Review of Current Developments and Prospects

**DOI:** 10.1021/acsomega.5c03269

**Published:** 2025-08-07

**Authors:** Abdullah Al Faysal, Setareh Dorreh, Beril S. Kaya, Ayşegül Gölcü

**Affiliations:** 52971 Istanbul Technical University, Faculty of Sciences and Letters, Department of Chemistry, Maslak, Istanbul 34469, Türkiye

## Abstract

The global struggle against infectious diseases represents a significant health issue that has persisted since ancient eras. Anti-infective agents are compounds that either prevent the spread of infectious pathogens or eradicate them. Halogens play a crucial role in the discovery and development of pharmaceuticals, as they are integral to synthesizing numerous drugs and medicinal compounds. These elements are vital across various categories of anti-infective agents and are found in many approved medications and promising lead compounds currently undergoing testing. The precise and swift identification of halogenated anti-infective agents is essential for clinical surveillance, food safety, and environmental research. Molecularly imprinted polymers (MIPs) have surfaced as a valuable resource for detecting these agents owing to their exceptional specificity, sensitivity, cost-effectiveness, and mobility. This review presents an extensive examination of the latest developments in the application of MIPs for detecting anti-infective agents. Despite the existing challenges, MIPs offer an economical and portable detection solution appropriate for on-site investigation across various fields. This review examines the fundamental operating principles, fabrication techniques, materials, and methodologies associated with MIPs. The following section delves into MIP-based sensors for precisely quantifying halogenated anti-infective agents across various sample types. Lastly, the review addresses the challenges faced in this field and outlines future directions. This comprehensive review aims to provide valuable insights that will aid in the enhancement of MIP sensors for clinically important applications in the future.

## Graphical abstract

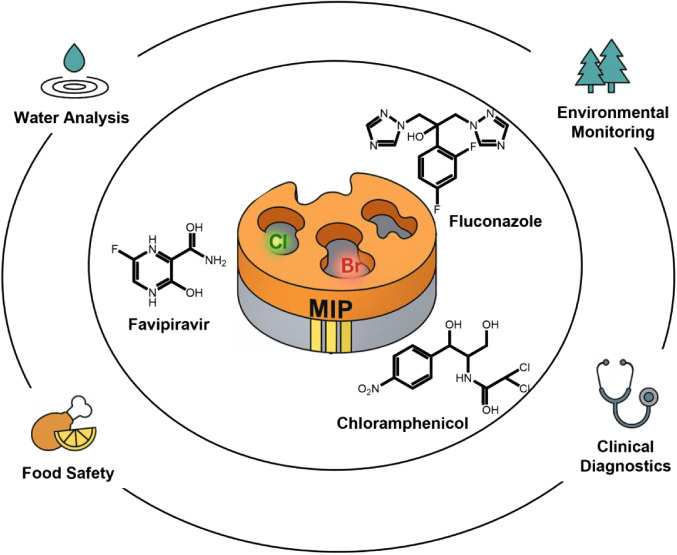

## 1 Introduction

Anti-infective agents are chemical substances that can either suppress the growth of microorganisms or induce bacterial cell death.^1,2^ Anti-infective medications are often categorized based on their primary microbiological targets rather than the illness or infection intended to treat. These are classified as antibacterial, antiviral, antifungal, and antiparasitic agents by pharmacopoeia and formularies. The majority of anti-infective medications are used to treat bacterial infections.^3^ Parasitic diseases are more prevalent in tropical and subtropical regions. Nevertheless, there is a small selection of medications that can be used to treat these parasitic infections, many of which have been used in clinical settings for many years.^4^

Halogen-containing compounds have gained significant attention in medicinal chemistry due to their remarkable contributions to drug development and therapeutic applications.^5^ Halogens, such as fluorine, chlorine, bromine, and iodine, enhance the pharmacological properties of drugs by participating in halogen bonding and facilitating specific interactions with biological targets.^5,6^ Furthermore, the electron density distribution in the σ-hole region of halogens facilitates covalent interactions, thereby increasing the affinity of drugs for their targets.^7^ This effect is important in improving drug efficacy within biological systems.^8^ Halogenation has proven effective in enhancing antibacterial and antiviral properties, as seen in halogenated polyphenols, resveratrols, and flavonoids.^5,9,10^ Halogenated indoles exhibit low toxicity, excellent bioavailability, and strong activity against drug-resistant microorganisms.^11,12^

Spectrophotometric methods, such as UV spectrophotometry, are widely used for detecting UV-active compounds like bedaquiline.^13^ Other approaches, including surface-enhanced Raman spectroscopy (SERS) for marbofloxacin detection using β-cyclodextrin-modified silver nanoparticles,^14^ chemiluminescence (CL) for pazufloxacin mesylate in serum and urine samples,^15^ and quantum dot (QDs)-based fluorescence imaging for pazufloxacin interaction studies,^16^ have expanded spectroscopic applications. Within 2 min, CdTe QDs in a dual-structure hydrogel coconstructed with poly­(vinyl alcohol) (PVA) and agarose (AG) demonstrated a quantifiable response to thiram at the micromolar level.^17^ Additionally, resveratrol (Res) was synthesized into resveratrol carbon quantum dots (CQD_Res_), and the antivirulence properties along with the underlying mechanisms of CQD_Res_ were elucidated for the first time.^18^ Furthermore, photosensitizers (PSs) demonstrating aggregation-induced emission (AIE) effects (AIE-PSs) were employed to enhance the photodynamic antibacterial interface.^19^ Chromatographic techniques, such as LC-MS/MS for bedaquiline in breast milk^20^ and fluoroquinolones in wastewater,^21^ and GC-MS for florfenicol in fish samples,^22^ provide precise and reliable detection. Methods like UPLC-MS/MS for tedizolid in rat plasma^23^ and HPLC for cephalosporins^24^ highlight their versatility. Electrochemical methods have advanced sensitivity, using materials like graphene oxide (GO) and rare earth metals, such as in the HV@GO sensor for sulfathiazole.^25^ Modified electrodes, including carbon paste (CPE) and glassy carbon electrode (GCE), have been applied for nadifloxacin detection using cyclic and differential pulse voltammetry.^26^ Other methods include ELISA for agents like chloramphenicol and thiamphenicol^27^ and nanoparticle-based approaches, such as glucose-reduced gold nanoparticles for pazufloxacin mesylate detection.^28^

Existing analytical techniques often struggle with challenges such as low sensitivity, poor selectivity, and difficulty in detecting trace biomolecules in complex samples. These issues make it hard to detect structurally similar molecules and handle low concentrations, which are common problems in clinical, environmental, and food analyses. Additionally, traditional methods tend to be costly and time-consuming.^29,30^ Molecularly imprinted polymers (MIPs) have emerged as biosensors to address these challenges. Their high selectivity, sensitivity, reusability, and mechanical stability make them effective tools for overcoming the limitations of traditional methods . MIPs offer cost-effective, robust alternatives for detecting specific molecules in complex biological or environmental samples, providing crucial benefits in applications requiring trace analysis or high specificity.^29^ The integration of MIPs into sensor systems, such as quartz crystal microbalance (QCM)-MIP^31^ and bulk acoustic wave (BAW) sensors,^32^ has enhanced their utility in detecting analytes like atropine^33^ and paracetamol.^32^ They have also been used in environmental monitoring, such as detecting bisphenol A,^30^ showcasing their versatility and significance.

This review thoroughly examines the significance of halogen-containing anti-infective agents and their role in combating infectious diseases. We discuss the unique pharmacological advantages that halogen atoms offer in drug design, their impact on enhancing drug–target interactions, and their applications across various categories, including antibacterial, antiviral, antifungal, and antiparasitic agents. Additionally, we explore advanced analytical and biosensor methods, particularly MIPs, which address the limitations of traditional detection techniques. This synthesis of existing knowledge highlights the critical role of halogenation in drug development and emphasizes innovative detection approaches, providing insights for future research and applications in medicinal chemistry.

## 2 Halogen-Containing Anti-infective Agents

Halogens have emerged as pivotal elements in the development of various antibiotics and their structural frameworks. Remarkably, a plethora of halogenated metabolites has been sourced from a diverse array of organisms, including microorganisms, algae, and a wide range of plants and animals.^5^ What sets halogens apart is their capacity for halogen bonding, a feature that significantly enhances their value in medicinal chemistry and drug development.^6^ Halogen bonds commonly form between halogen ligands and Lewis bases at critical binding sites. Furthermore, halogens demonstrate the ability to engage in covalent interactions, acting as both electrophiles and nucleophiles.^7^ The electron density in the σ-hole region determines the nature of these interactions. Due to their heavy atomic structure, halogens exhibit an uneven electron distribution. The three lone electron pairs create a cylindrical cloud with increased electron density around the atom, resulting in a positive electrostatic potential known as the σ-hole.^8^ This phenomenon is essential for boosting drug affinity within biological systems and is strategically leveraged to improve the effectiveness of drugs in therapeutic settings.^5^

The halogenated derivatives of polyphenolic compounds exhibit remarkable pharmacological benefits, particularly in antibacterial activity. Research has demonstrated that halogenated catechol significantly inhibits both Gram-positive and Gram-negative drug-resistant bacteria compared to its nonhalogenated form. Furthermore, studies reveal that halogenation amplifies the antibacterial effects of Magnolol, highlighting its potential.^5^ Notably, halogenated resveratrols have emerged as promising candidates in the fight against antimicrobial resistance. The incorporation of chlorine or bromine atoms into the aromatic ring enhances these compounds’ antimicrobial properties, increasing their lipophilicity and enabling them to effectively penetrate bacterial membranes and generate peroxyl radicals. Additionally, halogenated resveratrols interfere with ATP production and bacterial adhesion, thereby further showcasing their remarkable antivirulence capabilities.^9^

Halogenated flavonoids exhibit powerful antimicrobial properties against bacteria by promoting the release of intracellular substances, enhancing cell permeability to biocidal agents, and inducing notable changes in cell morphology.^10^ Furthermore, derivatives of halogenated indole are being effectively utilized as antibacterial agents against challenging antibiotic-resistant microorganisms.^5^ Extensive research through in vivo, in vitro, and in silico methods has confirmed that halogenated indoles possess low to mild toxicity levels, making them safer options for treatment.^11^ Additionally, halogenated trisindoles stand out for their exceptional drug-like characteristics, outstanding oral absorption, manageable toxicity, and impressive bioavailability.^12^

### 2.1 Antiparasitic Drugs

#### 2.1.1 Global Impact of Parasitic Diseases and Their Challenges

Infectious diseases can result from multiple organisms such as viruses, bacteria, parasites, and fungi in both humans and animals.^34,35^ Diseases caused by parasites continue to be a significant issue for humanity. Globally, they are increasingly the primary cause of chronic illnesses. Diseases spread quickly due to population migration, environmental contamination, and climate change. There is also evidence of parasites being more resistant to medications.^35,36^

#### 2.1.2 Classification of Parasites and the Role of Antiparasitic Medications

The medical community recognizes parasites as serious threats, categorizing them as protozoa, helminths, and arthropods. These organisms can lead to debilitating diseases or act as carriers for other dangerous pathogens.^34,37^ Antiparasitic medications are crucial in tackling infestations caused by these diverse organisms, particularly protozoa, with their complex life cycles that often involve multiple hosts, and helminths, with their intricate organ systems. Among the promising solutions are halogenated flavonoids, which exhibit robust antimicrobial properties against bacteria. They work by facilitating the release of intracellular substances, increasing cell permeability to biocidal agents and causing marked changes in cell morphology. Furthermore, halogenated indole derivatives are proving to be effective antibacterial agents against the formidable challenge of antibiotic-resistant microorganisms. Extensive research—spanning in vivo, in vitro, and in silico studies—demonstrates that halogenated indoles possess low to mild toxicity levels, making them safe and effective treatment options. These innovative compounds represent a powerful arsenal in the ongoing battle against bacterial infections, offering hope where conventional treatments may fail. Embracing these advancements can lead to more effective therapeutic strategies and improved patient outcomes.^37^

#### 2.1.3 Challenges and Efforts in Antiparasitic Drug Development

Despite the acute need for efficient antiparasitic drugs to treat populations in developing countries, pharmaceutical companies are generally not interested in researching new antiparasitic drugs. As a result, drugs invented in the last century continue to be used alone or in combination therapy. To address this gap, public–private partnerships have been initiated to support antiparasitic drug discovery programs, aiming to bridge the divide and develop more effective treatments for these vulnerable populations.^34,35^

#### 2.1.4 Halogen-Containing Antiparasitic Medications

The DrugBank Online database^38^ highlights an impressive selection of 144 antiparasitic medications that are crucial for effective treatment. A careful analysis of their chemical structures reveals that 42 of these powerful drugs incorporate halogen atoms within their molecular frameworks (as shown in Figure 1). This significant finding underscores the importance of halogenated compounds in the development of effective antiparasitic therapies.

**Figure 1:**
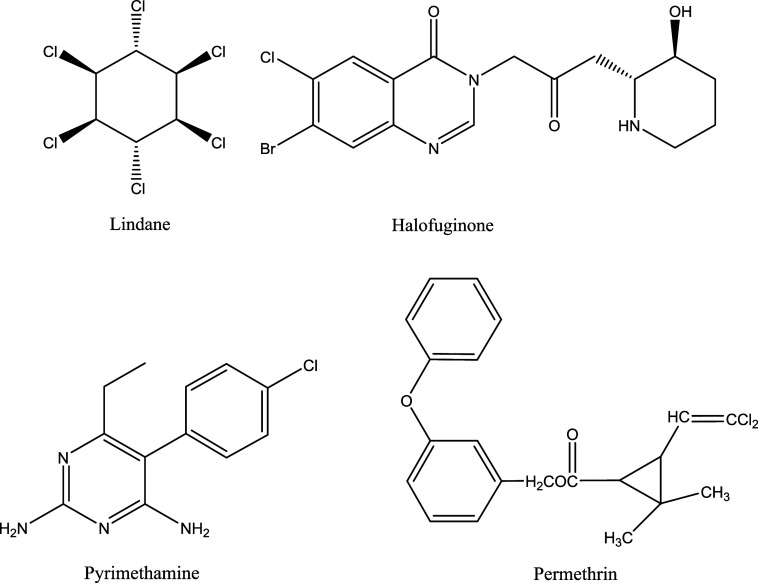
Structure of some of the halogen-containing antiparasitic drug molecules.

### 2.2 Antifungal Drugs

#### 2.2.1 The Dual Role of Fungi: Beneficial and Pathogenic Impacts

Fungi, including the intriguing bioluminescent mushrooms known as foxfire, are some of the most remarkable organisms on the planet. They play critical roles in sustaining ecosystems and profoundly impact human society. While many fungi are invaluable allies, contributing to the creation of cheese and alcohol, we must also recognize the looming threats posed by certain pathogenic species. These fungi can trigger severe infections, presenting significant global health risks that cannot be overlooked.^39^

#### 2.2.2 Global Burden of Invasive Fungal Infections

Invasive fungal infections claim an alarming 1.7 million lives each year, disproportionately affecting those who are immunocompromised due to chemotherapy, AIDS, and organ transplants. The ongoing global AIDS crisis, the rising use of medical implants, and improved survival rates have created a critical situation, contributing significantly to the increase in these dangerous infections. Awareness and proactive measures are essential to combat this growing threat.^40^

#### 2.2.3 Categories of Antifungal Drugs and Recent Advancements

When it comes to tackling fungal infections, our treatment options are restricted to just five categories of antifungals. However, only three of these have proven to be effective as standalone treatments. Caspofungin, micafungin, and anidulafungin represent a ground-breaking class of antifungals known as echinocandins, which are leading the charge in innovative antifungal therapy. Choosing these advanced medications can make a significant difference in effectively managing and treating fungal infections.^39,40^

#### 2.2.4 Antifungal Resistance: A Growing Concern

Despite being accessible, the excessive use of antifungals in human healthcare, agricultural protection, and veterinary treatment has led to worldwide resistance, especially against azoles.^39^ The widespread use of azoles in various sectors results in their release into the environment through wastewater, agricultural runoff, and waste disposal. This contamination harms nontarget organisms and accelerates the development of azole-resistant fungal strains, causing issues like algal growth inhibition, endocrine disruption in fish, and altered sex differentiation in frogs.^41^ The World Health Organization supports a Global Action Plan to combat antimicrobial resistance, promoting a One Health approach that integrates efforts across human and veterinary medicine, agriculture, and environmental sectors. The plan focuses on raising awareness and implementing measures to curb the spread of antifungal resistance.^42^

#### 2.2.5 Halogen-Containing Antifungal Medications

The DrugBank Online database^43^ identifies 85 antifungal agents that are essential for therapeutic applications. A thorough examination of their chemical structures reveals that 36 of these compounds contain halogen atoms, highlighting the potential significance and effectiveness of these unique molecular features in combating fungal infections (Figure 2).

**Figure 2:**
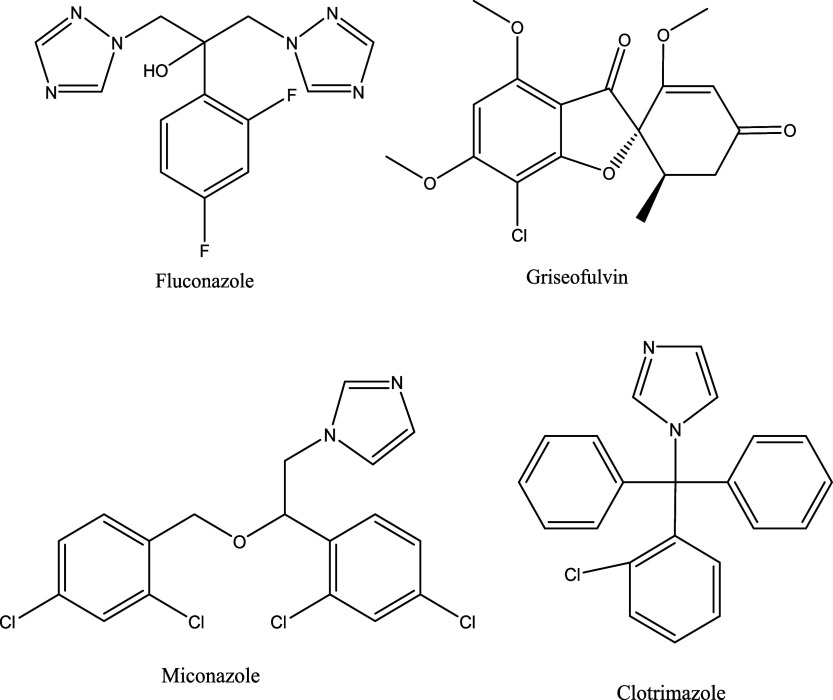
Structure of some of the halogen-containing antifungal drug molecules.

### 2.3 Antiviral Drugs

#### 2.3.1 Understanding Viral Pathogens: A Step toward Antiviral Development

Infectious diseases have posed a persistent threat to humanity throughout history, arising from various microorganisms, including bacteria, viruses, and fungi. Among these, viruses are particularly concerning because they are responsible for a vast array of diseases that can significantly impact human health. Unlike more complex organisms, such as fungi or protozoa, viruses possess a remarkably simple structure, which includes a protein coat, genetic material, viral enzymes, and occasionally a lipid envelope. Despite their small size, these agents can profoundly affect not only humans but also animals and plants, underscoring the importance of understanding and addressing viral infections.^44^

#### 2.3.2 Importance of Antiviral Drug Development

The development of antiviral drugs is vital, as viral infections have historically resulted in countless fatalities.^44,45^ The launch of the first antiviral medication, idoxuridine, in 1963 represented a monumental leap forward in the fight against viral diseases.^44^ Since then, numerous antiviral drugs have been developed, providing hope and relief to millions of people globally. Furthermore, the urgent need to address viral outbreaks and pandemics has driven rapid advancements in existing treatments and spurred the discovery of innovative antiviral therapies. The importance of continuing this progress cannot be overstated.^44,45^

#### 2.3.3 Mechanisms and Classes of Antiviral Drugs

Antiviral medications play a crucial role in fighting viral infections by effectively halting the growth and replication of viruses. Unlike antibiotics that target bacteria, these specialized treatments focus solely on viral threats. By either directly attacking the virus or disrupting the critical cellular factors on which it relies, antivirals make a significant impact on patient health. There are various antiviral drugs, including inhibitors that prevent virus attachment, entry, uncoating, and crucial processes such as polymerase activity, protease function, reverse transcriptase, and integrase. Choosing the right antiviral can improve health outcomes and provide a stronger defense against viral diseases.^44,45^

#### 2.3.4 Halogen-Containing Antiviral Medications

The DrugBank Online database^46^ features an impressive array of 216 antiviral drugs that serve vital therapeutic roles. A detailed examination of their chemical structures has uncovered that 66 of these essential medications incorporate halogen atoms, highlighting their unique molecular properties (Figure 3). This distinction emphasizes the significance of halogenated compounds in the development of effective antiviral treatments.

**Figure 3:**
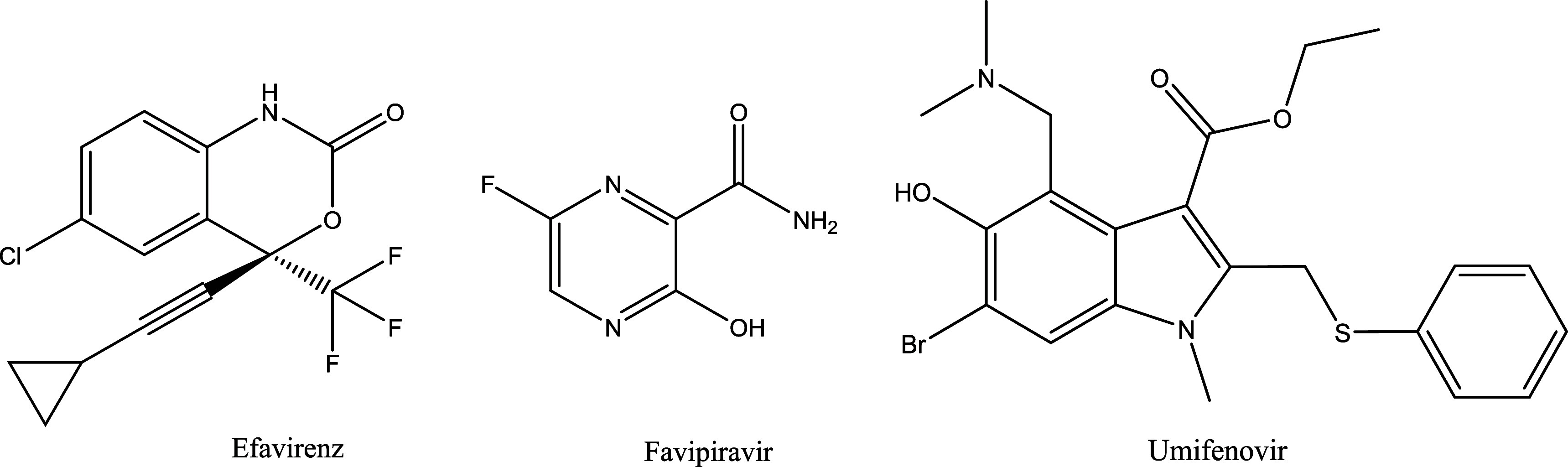
Structure of some of the halogen-containing antiviral drug molecules.

### 2.4 Antibacterial Drugs

#### 2.4.1 Historical Roots of Antibiotics: From Ancient Remedies to Modern Medicine

The discovery of antibiotics in the early 20th century has revolutionized medicine and saved many lives. The use of antimicrobial agents to combat illnesses has a long history that dates back to ancient societies that utilized a variety of natural extracts for their therapeutic properties.^47^ Antibiotic-producing microorganisms have been utilized to prevent disease for thousands of years. More than 2000 years ago, traditional treatments like moldy bread poultices were used to treat wounds in areas like Serbia, China, Greece, and Egypt.^48^

#### 2.4.2 The Birth of Modern Antibiotics and Their Transformative Impact

The term “antibiotics” emerged from the pioneering research of American microbiologist Selman Waksman and his team, who were the first to isolate chemical compounds from microorganisms that could inhibit the growth of other microbes.^47^ The first antibiotic, salvarsan, was introduced in 1910. Antibiotics have made a major impact on modern healthcare during the past century, helping to extend the average human lifespan by 23 years. Antibiotics have made it possible for many modern medical operations, including cancer treatments, organ transplants, and open-heart surgeries, in addition to treating infectious infections.^48^

#### 2.4.3 Mechanisms of Action in Antibiotics

These medications play a crucial role in targeting the biochemistry and physiology of microbial cells, effectively inhibiting their growth or leading to their destruction. Some antibiotics effectively dismantle the cell walls or membranes of bacteria, whereas others disrupt protein synthesis by binding to ribosomal units. This interruption not only halts the bacteria’s ability to function but also enhances the effectiveness of treatment, making antibiotics essential in our fight against bacterial infections.^47^

#### 2.4.4 The Growing Threat of Antimicrobial Resistance

Unfortunately, the potential for developing resistance to any therapeutic agent undermines their effectiveness.^47^ The inappropriate use of these essential drugs has caused a rapid increase in antimicrobial resistance, making some infections nearly impossible to treat.^48^

#### 2.4.5 Halogen-Containing Antibacterial Medications

According to the DrugBank Online database,^49^ a total of 369 antibacterial drugs are currently utilized for therapeutic purposes. A detailed examination of their chemical structures shows that 78 of these medications incorporate halogen atoms, highlighting the significance of these elements in their design and function (refer to Figure 4).

**Figure 4:**
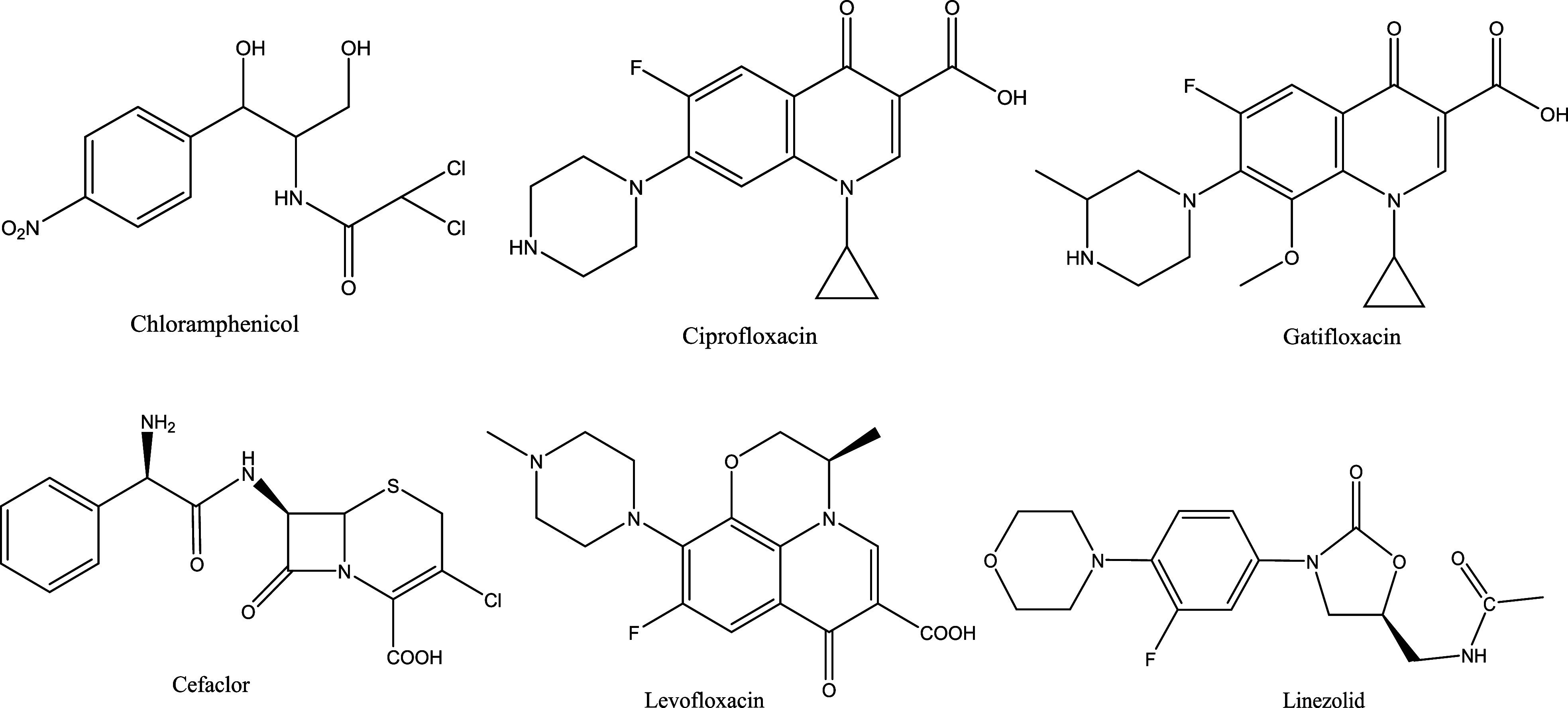
Structure of some of the halogen-containing antibacterial drug molecules.

## 3 Current Methods for Halogen-Containing Anti-infective Agents’ Detection

### 3.1 Non-Electrochemical Techniques

UV-active agents like bedaquiline (BDQ) can be detected by UV spectrophotometry due to their chromophore groups^13^ while derivatization is required for UV-inactive ones like glibenclamide and eflornithine using 9,10-phenanthraquinone (PQ)^50^ and O-phthalaldehyde/2-mercaptoethanol (OPA-MCE), respectively.^51^ Lumichrome-based probes have been applied for chlortetracycline^52^ detection under various conditions.^53^ Nanotechnology-enhanced methods include surface-enhanced Raman Spectroscopy (SERS) using β-cyclodextrin modified silver nanoparticles (β-CD-AgNPs) for marbofloxacin^14^ and Chemiluminescence (CL)-based flow injection for pazufloxacin.^15^ Quantum dots interacting with pazufloxacin have been studied for fluorescence changes.^54^ Chromatographic systems like liquid chromatography-tandem mass spectrometry (LC-MS/MS), ultraperformance liquid chromatography-tandem mass spectrometry (UPLC-MS/MS), gas chromatography mass spectrometry (GC-MS), and high-performance liquid chromatography (HPLC) have been used for various agents including BDQ,^20^ florfenicol (FF),^22^ cephalosporin,^24^ and tedizolid.^55^

Capillary electrophoresis (CE) and reversed-phase high-performance liquid chromatography (RP-HPLC) were applied for FF,^56^ and pazufloxacin was also detected with CE-electroluminescence (ECL).^57^ Micellar liquid chromatography (MLC) using sodium dodecyl sulfate (SDS) as a surfactant, enabled the detection of danofloxacin and marbofloxacin^58^ while fluoroquinolones in honey were separated using Fe^3+^-affinity columns^59^ and nadifloxacin in creams via high-performance thin-layer chromatography (HPTLC).^60^ Enzyme-linked immunosorbent assay (ELISA) was used for florfenicol amine (FFA),^61^ chloramphenicol (CAP), and thiamphenicol (TAP)^27^ and indirect competitive ic-ELISA for adriamycin.^62^ Accelerated solvent extraction (ASE) combined with ultraperformance liquid chromatography-fluorescence (UPLC-FL) detected residues in meat.^63^ A calorimetric assay using gold nanoparticles (GNPs)^28^ and glucose as a reducing agent targeted pazufloxacin.^64^ Metal-free agar plates with *Bacillus subtilis* enabled the screening of fluoroquinolones in honey followed by LC/FL.^65^ A triple-mode aptamer sensor using Pt and Fe/Co-MOF (metal–organic framework) was developed for sarafloxacin.^66^

### 3.2 Electrochemical Techniques

Graphene-based materials are widely used for sensor fabrication due to their excellent conductivity^67^ and stability.^68^ Holmium vanadate shielded by graphene oxide (HV@GO) sensor detected sulfathiazole electrochemically^25^ while doxycycline and chlortetracycline were analyzed by voltammetry using pulse amperometry.^69^ Electrospun polyacrylonitrile (PAN)/covalent organic framework TpPa–1 nanofiber enabled quinolone^70^ detection in food samples.^71^ Sarafloxacin hydrochloride-sodium tetraphenylborate (SARA-TPB) sensor for SARA was characterized by advanced microscopy techniques.^72^ Nadifloxacin detection was achieved by a modified carbon-paste electrode (CPE) with carbon nanomaterials^73^ and a glassy carbon electrode (GCE) with multiwalled carbon nanotubes and TiO_2_ nanoparticles (MWCNT-TiO_2_), characterized by scanning electron microscopy (SEM), cyclic voltammetry (CV), and electrochemical impedance spectroscopy (EIS).^26^

## 4 Molecularly Imprinted Polymer (MIP)

### 4.1 Principle

The imprinting technique is included in the literature as a method that aims to create selective binding sites for a specific molecule or ion in a polymer matrix.^74^ Molecular imprinting is one of the methods for developing high-sensitivity materials for the desired purpose by creating specific recognition and binding sites for the target molecule by using this technique. The high amount of cross-linkers in their structures makes them durable for the molecule and increases the binding capacity of the polymer. The name is given according to the mold used in printing. In this context, if molecules such as organic or biological structural elements are used as molds, it is called molecular imprinting, and the polymer on which the printing is made is called MIP.^75^ In short, it can be summarized as a template-molecule-specific polymer synthesis process. This subject was first introduced to the literature by Wulf and Sarhan in 1972.^76^ However, Dickey’s goal of synthesizing selective silk gel specific to dye molecules using Linus Puling’s antibody production method in the 1950s can be considered the cornerstone in obtaining the first imprinted polymer.^77^ Scientists’ work on imprinted polymers is increasing every year, as shown in Figure 5 below.

**Figure 5:**
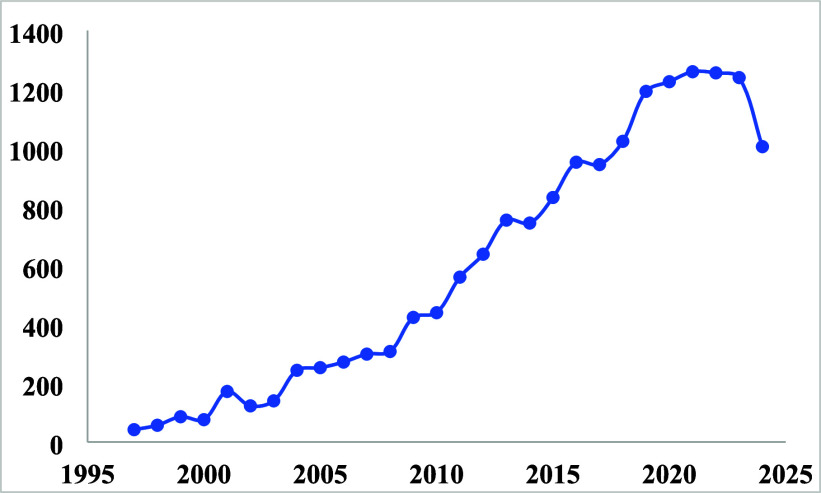
Number of publications by year according to the Web of Science.

The ability of MIP technology to provide materials with defined selectivity, durability, and easy preparation gives them hope. At the same time, it has numerous advantages, such as being produced at low cost, being reusable, having reusable, and having high mechanical and heat resistance. Such applications benefit many areas, from pharmaceutical analytics to environmental analysis, and offer long-term use.^78^

### 4.2 Fabrication Process

In light of all this information, three steps are needed to perform the MIP method. These steps are precomplexation, polymerization, and removal of the target molecule.^79^ The precomplexation stage begins with the formation of a complex between the monomer, which can be polymerized and has a suitable functional structure, and the analyte, called the target molecule, through covalent or noncovalent interactions. In this process, the monomer surrounds the template molecule and accelerates the polymerization process in an orderly and increasing manner. In the polymerization stage, the preformed monomer–template complex is subjected to the polymerization process by adding cross-linkers to the medium. In this process, functional monomers form a matrix around the target molecule, and thanks to the cross-linkers, the polymer gains mechanical strength and three-dimensional structure. In the final stage, the template molecule in the polymer must be removed from the environment. In this way, cavities are formed in the polymer structure according to the target molecule’s shape, size, and chemical properties. Finally, when the solution containing the template molecule is applied to this imprinted polymer, the template molecule reattaches, thanks to the presence of recognition sites. The main factor that provides selective binding to the target molecule is this issue. There are three types of imprinting mechanisms in this technology; the most widely used technique is noncovalent imprinting. Template and functional monomer binding occur as a result of weak interactions, such as electrostatic forces and van der Waals forces. It is also quite easy to remove the template from the polymer prepared by this method.^80^ In covalent imprinting, the monomer and template interact with each other via strong covalent bonds, making it difficult for the template to be removed from the polymer. Semicovalent printing technology, which includes the advantages of these two methods, has similar properties to the noncovalent method.^81^ The most important thing to note is that the template molecule must exhibit physical or chemical interactions with the monomer. To ensure these interactions, it is important that the template molecule has an appropriate solubility during the polymerization process. In addition, the availability of these materials at a low cost is a critical factor in the efficiency and economy of the synthesis process. These interactions between the template molecule and the monomer will determine the properties of the polymer and increase its binding capacity with the target molecule.^82^ Monomer selection plays a critical role in the MIP synthesis. This is mainly because it interacts effectively with the target molecule and forms a prepolymerization complex with the template molecule. To do this, functional groups compatible with the template molecule must be selected and a strong bond between molecules must be provided. Additionally, using a support matrix increases the specificity of MIP polymers.^83^ When the studies are examined, the most commonly used monomers are methacrylic acid (MAA), 4-vinylpyridine, 2-hydroxyl methacrylate (HEMA).^78^ The cross-linking agent is responsible for the formation of the polymer network and giving hardness to the polymer.^84^ It has an important place in determining the morphology of the polymer matrix. It regulates whether the matrix is gel-type, macroporous, or microgel. The cross-linker and the polymer must be in a suitable stoichiometric balance with each other. Using this agent in low or high molar ratios may cause various undesirable situations. Using less than necessary will result in the binding sites of the template molecules being too close to each other and the binding sites of the target molecule being physically inaccessible to neighboring sites. The use of large amounts leads to noncovalent interaction of the binders with the monomer or template molecules.^85^ Divinylbenzene and ethylene glycol dimethacrylate are commonly used as cross-linkers. The main component that starts the polymerization process is called the initiator, and this initiator allows the monomers to combine to form a polymer network. The initiator is added to the process in a controlled manner so that the structure and properties of the polymer are directed as desired. The amount of initiator is usually kept quite low because the initiator does not only play a role in initiating the reaction. The initiator, which is used in a much smaller amount compared to the monomer, is used effectively, and thus the formation of undesirable byproducts is minimized.^86^ The selection of initiators plays an active role not only in initiating the reaction but also in forming the physicochemical properties of the polymer. Generally preferred initiators are 2,2-azobis­(2,4-dimethylvaleronitrile) (AIBN) and 2,2-azobis­(2,4-dimethylvaleronitrile) (ADVN).^81^ In MIP technology, in addition to all of the basic elements mentioned above, the solvent also constitutes a critical part of the process. The template increases the binding speed of the molecule, allowing interactions to occur more effectively. The solvent chosen for MIP should be of high solubility and usually of low dielectric constant in order to preserve the noncovalent interactions between the template molecule and the functional monomer.^87^

Numerous electrochemical MIP-based sensors have been developed to detect halogenated anti-infective drugs, following shared design principles guided by the physicochemical structure of the analytes. Common polymerization methods include precipitation polymerization,^88^ mini-emulsion polymerization,^89^ and electropolymerization.^90–92^ Functional monomers such as methacrylic acid and acrylamide are frequently used to create highly selective binding cavities.

### 4.3 Extraction

In molecular imprinting polymers, the target molecule is removed by extraction. This process separates the target molecule from the matrix, leaving only analyte-specific cavities. These cavities allow MIPs to bind selectively. The target molecule is usually removed with solvents. The purpose of the solvent here is to break the interaction with the target molecule and separate it from the polymer. In this context, there are certain criteria that the solvent must have. Selecting solvents with low polarity and high solvency capacity will ensure efficient separation from the polymer.^93^ MIPs synthesized specifically for a particular molecule have various facilitating effects such as the ability to selectively capture the target molecule and its traceability; therefore, they are frequently used in extraction processes. MIPs can be used in analytical chemistry, especially for solid phase extraction (SPE).^94^ It acts as a sorbent in this technique. MISPE consists of the same steps as SPE in principle. After the sorbent is conditioned, sample loading occurs and elution is obtained from washing. The only difference is that the sample is passed through MIP sorbents during loading.^93^ The first application of MISPE in the literature was provided by the extraction of the drug called pentamidine, which is used in the treatment of AIDS disease.^95^ Different drugs were extracted by combining the same system with HPLC.^96^ In addition, MIP technology is also used in Solid Phase Microextraction (SPME), Stirrer Bar Sorbing Extraction (SBSE), and Matrix Solid Phase Distribution (MSPD) techniques. Polymethylsiloxane (PDMS) is traditionally used in SPME, but using MIP instead increases fiber selectivity. It is prepared by the bulk polymerization method.^97^ In the SBSE technique, MIPs separate molecules with low concentration and complex matrices.^98^

### 4.4 Types of MIP Sensors

With the advancement of technology and the effects of the increasing population in recent years, new demands have emerged in many areas such as clinical diagnosis and environmental or food analysis. The variety of features it provides, such as high selectivity and sensitivity and the detection of the desired substance in almost any matrix, makes this technique a pioneer in many areas such as determining toxic substances in environmental analysis and monitoring contaminants in food inspections. Particularly, the large and complex structures of biomolecules and the low concentrations of these molecules are among the factors that constitute the difficulties faced by bioanalytical researchers. Therefore, using MIPs as biosensors is rapidly becoming widespread to overcome these difficulties.^29^

#### 4.4.1 Electrode-Based MIP Sensors

Electrode-based MIP sensors represent one of the most widely studied and applied classes of molecularly imprinted sensors. In this configuration, the imprinted polymer is directly integrated onto the surface of an electrode, such as screen-printed carbon electrodes (SPCE), glassy carbon electrodes (GCE), or carbon paste electrodes (CPE), to ensure specific recognition of the target molecule. The performance of these sensors largely depends on the effective binding of the analyte within the MIP cavities and the efficient electron transfer between the MIP layer and the electrode surface. It has been observed that the obtained sensor response is directly dependent on the integration level of the MIP and the transduction element and the electrical communication efficiency between them.^99^ Atropine was detected in human serum and urine using this sensor system.^33^ Various modification strategies have been employed to enhance sensitivity and selectivity, including the incorporation of nanomaterials such as gold nanoparticles (AuNP), reduced graphene oxide (rGO), carbon nanotubes (CNT), and metal–organic frameworks (MOFs) to improve conductivity and surface area.^100^ Additionally, detection of 8-lactoglobulin (8-LG) was achieved with an MIP sensor developed using reduced graphene oxide (rGO) and gold nanoparticles (AuNp).^101^ A carbon paste electrode (CPE) modified with a magnetic molecularly imprinted polymer (MMIP) was developed for the recognition of amoxicillin and other beta-lactam antibiotics.^102^

To enhance sensitivity, nanomaterials like gold nanoparticles (AuNPs),^103,104^ reduced graphene oxide (rGO),^91,105^ and metal–organic frameworks (MOFs)^106,107^ are incorporated to increase conductivity and surface area. Other materials, such as TiO_2_ nanotubes,^108^ have been employed in photoelectrochemical sensors for analytes.

Electrochemical techniques such as differential pulse voltammetry (DPV),^90,104^ electrochemical impedance spectroscopy (EIS),^105^ and photoelectrochemistry (PEC),^108,109^ are selected according to matrix complexity and target sensitivity. These design choices are often inspired by the need to achieve ultratrace detection in complex matrices such as serum, plasma, food, or environmental samples and to differentiate structurally similar compounds.

These trends reveal that recent MIP sensor development is highly modular, where polymer structure, nanomaterial selection, and transduction methods are adapted to the properties of each halogenated anti-infective agent to enable selective and sensitive detection in complex real samples.

#### 4.4.2 Acoustic MIP Sensors

Surface acoustic wave (SAW) sensors have attracted attention due to their high sensitivity, low power consumption, fast response time, and suitability for miniaturization.^110^ The integration of molecularly imprinted polymers (MIPs) with SAW technology provides selective recognition capabilities for target analytes, enabling highly sensitive gas-phase detection. The first MIP-based SAW sensor was developed in 1998 for o-dimethylbenzene, demonstrating a detection limit as low as 4.5 mg/m^3^ and excellent selectivity.^111^ Advances in self-assembled monolayers (SAMs) and supramolecular architectures, such as calixarene- or cyclodextrin-functionalized films, have further improved the chemical recognition of SAW sensors.^112^ For example, β-cyclodextrin derivatives were used to design a SAW sensor with enhanced sensitivity toward organophosphorus agents like sarin, exploiting host–guest interactions and steric complementarity.^113^ Acoustic sensors such as quartz crystal microbalance (QCM) and bulk acoustic wave (BAW) sensors have been developed by integrating MIPs onto piezoelectric surfaces. In one study, paracetamol was detected in urine by imprinting the analyte on a piezoelectric quartz crystal-coated surface.^32^ A QCM-MIP sensor was also employed for the detection of the potential carcinogen daminozide in food samples.^114^

#### 4.4.3 Thermoresponsive MIP Sensor

Thermoresponsive molecularly imprinted polymers (MIPs) have emerged as a promising class of smart materials with selective recognition abilities combined with a temperature-triggered functionality. They target temperature changes for the analysis of the interaction between the target molecule and the binding sites of MIP. This structured MIP sensor designed to detect fructose valine obtained a 40-fold higher signal than the control polymer.^115^ It has also been used to detect the chemical bisphenol A (BPA), which is used in producing durable and transparent plastics and is not only an endocrine disruptor but also quite harmful to the environment.^30^ In one study, a thermoresponsive MIP was synthesized using a pseudotemplate strategy for the selective adsorption and temperature-regulated release of the anticancer drug methotrexate (MTX).^112^ This approach not only allowed specific recognition of MTX but also enabled its controlled delivery upon temperature change, a key advantage for drug delivery systems (DDS) aiming to minimize side effects and enhance targeting.^116^ Compared to conventional MIPs, the incorporation of thermal responsiveness provides additional functionality critical in the biomedical and pharmaceutical fields.

#### 4.4.4 Chiral and Enantiomer-Selective MIP Sensors

MIP technology has also been applied in enantioselective recognition. These sensors differentiate between enantiomers that share the same molecular formula but differ in spatial configuration.^117^ Despite their potential, MIP-based chiral electrochemical sensors still face challenges in achieving complete enantioselectivity, largely due to the limited separation power of direct electrochemical detection compared to chromatographic techniques.^118^ The effectiveness of chiral recognition depends on the combination of multiple noncovalent interactions that cooperatively contribute to stereoselectivity. For instance, a methacrylate-based R­(+)-atenolol (ATNL)-imprinted MIP has been employed as a chiral recognition platform, enabling both direct and indirect electrochemical detection of ATNL enantiomers, thereby demonstrating the molecular recognition mechanisms within the polymer matrix.^119^

In a related study, propranolol enantiomers were targeted using a molecularly imprinted polymer synthesized via suspension polymerization and thiol-maleimide click chemistry, demonstrating high enantioselectivity and binding affinity, thus confirming the effectiveness of MIP-based approaches for chiral separation.^120^

#### 4.4.5 Impedimetric and Nanofiber-Based MIP Sensors

Impedimetric MIP sensors are electrochemical devices that detect target molecules by measuring changes in impedance resulting from selective binding to molecularly imprinted polymer layers.^121^ Also, nanofibers significantly boost the MIP sensor performance by enabling efficient analyte transport and high binding site accessibility. For instance, an impedimetric MIP sensor was developed for the detection of zearalenone (ZEN), a carcinogenic mycotoxin, using polypyrrole-based imprinting on screen-printed electrodes. The sensor demonstrated high selectivity and sensitivity within a linear range of 1–500 pM, with a detection limit as low as 1 pM. The method was successfully applied to grain samples such as corn, rice, and wheat, showing excellent recovery rates and minimal cross-reactivity with structurally related toxins.^122^ Detection of dengue infection has been made possible with an MIP-based impedimetric sensor. This process was achieved by modifying the screen-printed carbon electrode (SPCE) with polysulfone nanofibers.^123^ The electrode prepared using reduced graphene oxide (rGO) with gold nanoparticles (AuNp) is included in the literature as an effective MIP sensor for the determination of 8-lactoglobulin (8-LG). For the selective recognition of amoxicillin and beta-lactam antibiotics, the carbon paste electrode (CPE) was modified with a magnetically molecularly imprinted polymer (MMIP). Polypyrrole (PPy) was functionalized with MIP for the molecular recognition of glyphosate (Gly), which is used as an herbicide.^124^ Another example featured a wearable electrochemical MIP sensor incorporating a gold nanoparticle-decorated 3D carbon nanofiber membrane (GnPs@CnFM) as the sensing matrix and a nanofiber-based microfluidic layer for efficient sweat transport, enabling real-time and selective cortisol detection.^125^

## 5 MIP Application for Detection of Halogen-Containing Anti-infective Agents

### 5.1 Antiparasitic Drugs

The rapid growth of tourism, combined with increased population mobility and shifts in global civilization, has led to an alarming rise in parasitic diseases in regions where they were once absent. Climate change further exacerbates this issue, resulting in a surge of parasitic infections. Despite these pressing challenges, pharmaceutical companies often neglect the development of new antiparasitic treatments. Consequently, we find ourselves relying on medications that were developed over a century ago, using them as standalone solutions or in combination with other drugs. It is vital to accurately and precisely identify these last-resort treatments.^35^ To aid in this critical effort, a comprehensive compilation of halogen-containing antiparasitic agents and their determination methods is provided in Table 1.

**Table 1:** Determination of Halogen-Containing Antiparasitic Drug Molecules Employing Different Types of MIP-Based Electrochemical Sensors^a^

Method	Analyte	Technique	Linear Range	LOD	Sample	Recovery	ref.
PoPD@TiO_2_ NTs	Lindane	PEC	0.1–10 μmol L^–1^	0.03 μmol L^–1^	Standard solution	95.5–104.8%	^108^
MWCNT-MIP	Lindane	Potentiometric	1 × 10^–9^–1 × 10^–3^ M	1.0 × 10^–10^ M	Water and vegetable	96.00–99.33%	^126^
MISPE-HPLC	Permethrin	HPLC–UV	20–120 μg/L	5.72 μg/L	Urine	93.01–97.14%	^127^
MISPE-GC-MS	Permethrin	GC-MS		3.1 μg kg^–1^	Food	99.3–126%	^128^
MMIP-HPLC	Permethrin	HPLC–UV	0.025–5.0	0.0068	Fruit	82.4–101.7%	^129^
MISPE-GC	Permethrin	GC	0.002–0.2 μg mL^–1^	0.009 μg mL^–1^	Honey	94.1–106.2%	^130^
OR-imp@PDA/ERGO/GCE	Ornidazole	DPCSV	1.5 × 10^–9^–2.0 × 10^–7^|M	1.1 × 10^–9^|M	Tablet	97.94–101.87%	^91^
B-MIHF/CPE	Ornidazole	DPV	6.0 × 10^–8^–3.5 × 10^–4^ mol L^–1^	1.1 × 10^–8^ mol L^–1^	Pharmaceutical and biological	96.00–108.00%	^131^
GQD-SMIP	Ornidazole	fluorescence	0.75 to 30 μM	0.24 μM	Plasma	89.60–119.26%	^132^
MIP monolith	Ornidazole	pCEC	0.80–100 μg/mL	0.13 μg/mL	Water and milk	93.6%	^133^
MIP-MCL	Coumaphos	CL	-	1.84 pg/mL	Milk	88.3%	^134^
Paper-based MIP	Dichlorvos	CL	3–1000 ng/mL	0.8 ng/mL	Vegetables	-	^135^
MISPE–HPLC	Dichlorvos	HPLC-UV	0.5–500 μg L^–1^	94.8 ng L^–1^	Vegetables	82.1–94.0%	^136^
QDs-MIMS	Dichlorvos	Fluorescence	5–25 μg/L	1.27 μg/L	Vegetables	87.4–101%	^137^
MISPE–GC	Dichlorvos	GC	0.05–10.0 mg/L	0.76 μg/L	Vegetables	92.25%	^138^
MISPE–GC	Dichlorvos	GC	0.001–10 mg/L	0.21 μg/kg	Vegetables	91.38%	^139^
MISPE-HPLC	Lumefantrine	HPLC-UV	50–10,000 ng mL^–1^	4.97 ng mL^–1^	Plasma	83.68–85.42%	^88^
Ni@MIL-100(Fe)@MIP	Hydroxychloroquine	HPLC-UV	1–300 μg L^–1^	0.2 μg L^–1^	Plasma	96–103%	^107^
PPy-MIP	Halofuginone	DPV	7.5 × 10^–9^–1.0 × 10^–5^ M	2.5 × 10^–9^ M	Chicken meat	97.40–103.25%	^140^
MIP-BAW	Pyrimethamine	BAW	6.0 × 10^–7^–1.0 × 10^–4^ M	2.0 × 10^–7^ M	Serum and urine	93.5–107.8%	^141^
Core–shell MIP	Pyrimethamine	HPLC	0.25–10.0 mg/L	0.1 mg/L	Fish and plasma	83.6–95.4%	^142^

a BAW = bulk acoustic wave; B-MIHF = boron-embedded molecularly imprinted hybrid film; CL = chemiluminescence; CPE = carbon paste electrode; DPCSV = differential pulse cathodic stripping voltammetry; ERGO = electrochemically reduced graphene oxide; GQD-SMIP = graphene-quantum-dot-embedded silica molecular imprinted polymer; HPLC = high-performance liquid chromatography; MCL = microtitration CL; MIL-100­(Fe) = Material Institute Lavoisier; MISPE = molecularly imprinted solid phase extraction; MMIPs = magnetic molecularly imprinted polymers; MWCNTs = multiwall carbon nanotubes; OR = ornidazole; pCEC = pressurized capillary electrochromatography; PDA = molecularly imprinted polydopamine; PEC = photoelectrochemical; PoPD = poly­(o-phenylenediamine); Ppy = polypyrrole; QDs-MIMS = quantum dots encapsulated molecularly imprinted mesoporous silica; TiO_2_ NTs = titanium dioxide nanotubes.

An advanced MIP specifically designed for lumefantrine was developed and validated by Silva et al.^88^ The synthesis of the polymers was achieved through precipitation polymerization, while chemometric techniques were utilized to optimize the synthetic parameters systematically. Lumefantrine was measured in human blood specimens from healthy participants using the established technique. The recoveries ranged from 83.68% to 85.42%, and the approach passed all validation criteria. Furthermore, it exhibited linearity across a concentration range of 50 to 10,000 ng mL^–1^. The theoretical LOD and LOQ were determined to be 4.97 ng mL^–1^ and 15.04 ng mL^–1^, respectively. The developed MISPE-HPLC-UV technique is a quick, highly sensitive, selective, and cost-effective way to monitor patients receiving lumefantrine medication. A selective photoelectrochemical sensor was developed by Wang et al.^108^ using molecularly imprinted PoPD film as recognition elements, integrated into vertically aligned TiO_2_ nanotubes (Figure 6). With a detection limit (LOD) of 0.03 μmol L^–1^, the photocurrent level rose linearly with the lindane concentration under ideal circumstances (0.1–10 μmol L^–1^). Five standard lindane solution samples were assayed in order to assess the PEC sensor’s analytical dependability and possible applications. The findings showed that the method’s reliability was satisfactory, with the relative standard deviation (RSD) being less than 5.0% and the recoveries falling between 95.5% and 104.8%. The MIP-based PoPD@TiO_2_ NTs PEC sensor presents significant potential for effective monitoring of lindane, characterized by a low detection limit and rapid detection time. A novel sensing platform based on MIPs has been developed for the electrochemical detection of ornidazole (OR) as reported by Güney.^91^ Dopamine was electropolymerized on a glassy carbon electrode that had been altered by electrochemically reduced graphene oxide to create the sensor (Figure 7). The sensor exhibited a linear analytical response to OR concentrations ranging from 1.5 × 10^–9^ M to 1.0 × 10^–8^ M and from 1.0 × 10^–8^ M to 2.0 × 10^–7^ M, with a detection limit established at 1.1 × 10^–9^ M. The designed sensor produced good results when used to assess OR in the drug sample. This innovative sensor holds considerable promise for routine analyses aimed at detecting very low concentrations of OR in various samples.

**Figure 6:**
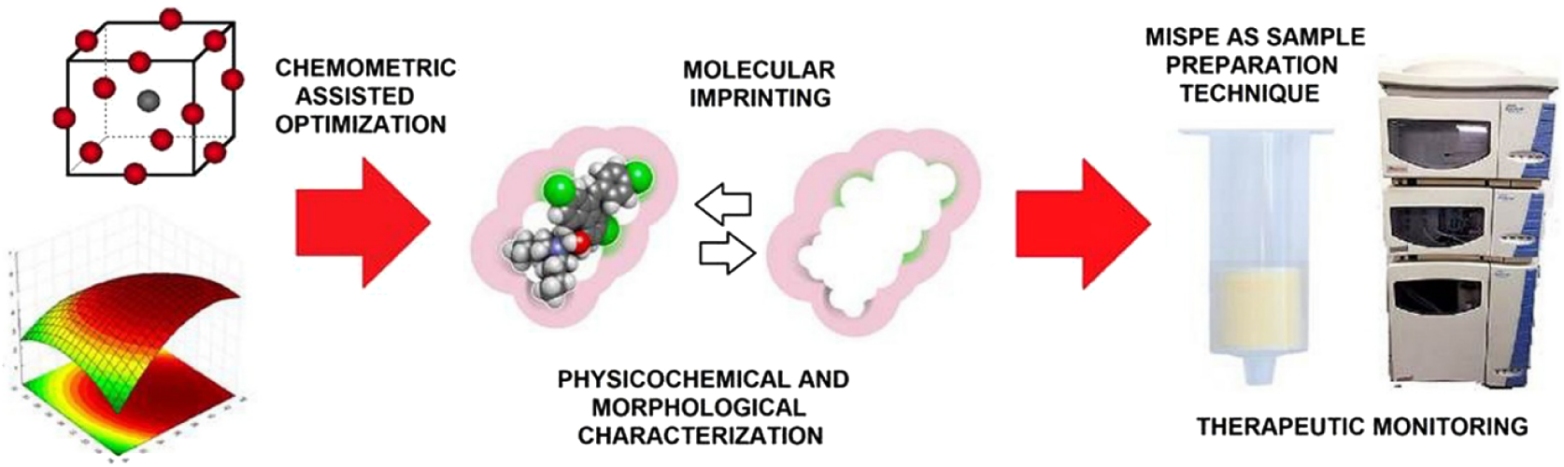
Creation and optimization of an MISPE-HPLC-UV technique for lumefantrine measurement. Reproduced from ref 88, with permission from Elsevier.

**Figure 7:**
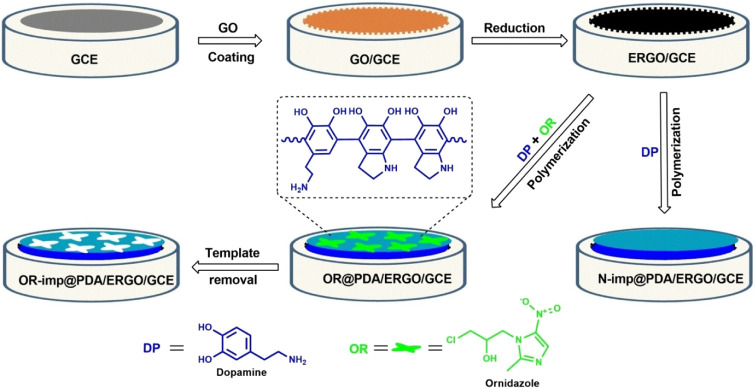
Synthesis of OR-imp@PDA/ERGO/GCE and N-imp@PDA/ERGO/GCE. Reproduced from ref 91, with permission from Wiley.

### 5.2 Antifungal Drugs

Fungal infections represent significant medical challenges characterized by high mortality rates even with appropriate therapeutic interventions. Compounding this issue is the emergence of acquired resistance to antifungal agents alongside the inherent resistance observed in certain fungal species. Presently, the market is limited to a small selection of antifungal agents that belong to a few distinct classes, each exhibiting varied mechanisms of action. This situation underscores the pressing necessity for the development of novel antifungal compounds and classes.^143^ In response to this urgent need, pharmaceutical companies are actively engaged in the research and development of new antifungal medications. This review aims to provide an overview of the various methodologies employed to investigate halogen-containing antifungal agents currently available, utilizing the MIP technique as a focal point (Table 2).

**Table 2:** Determination of Halogen-Containing Antifungal Drug Molecules Employing Different Types of MIP-Based Electrochemical Sensors^a^

Method	Analyte	Technique	Linear Range	LOD	Sample	Recovery	ref.
MIP–SPE	Fluconazole	UPLC–MS	1.17 × 10^–5^ to 1.27 × 10^–4^ mM	1.63 × 10^–10^ mM	Capsule	91%	^92^
DCE-DMIP	Clotrimazole	HPLC	0.1–20 μg L^–1^	0.027 μg L^–1^	Water	91.3–95%	^144^
DMIM-MSPD	Clotrimazole	HPLC	0.25–25 μg g^–1^	0.036 μg g^–1^	Fish	97.9–101.5%	^145^
PS-*co*-PMAA@PMIP	Griseofulvin	UV	0–2.8 × 10^–5^ mol L^–1^	2.8 × 10^–7^ mol L^–1^	Milk	100–107.1%	^146^
SMIP-HPLC	Griseofulvin	HPLC	0.1–50 μg/mL	0.02 μg/mL	Plasma	90.7–97.7%	^147^
SMIP-DSPE	Griseofulvin	HPLC	0.1–100 μg/mL	0.01 μg/mL	Water	91.6–98.8%	^148^
MIP-PT-μ-SPE	Ketoconazole	HPLC	6.25–650 ng mL^–1^	6.25 ng mL^–1^	Urine	99.24–113.37%	^149^
DCE-DMIP	Miconazole	HPLC	0.1–20 μg L^–1^	0.023 μg L^–1^	Water	88.6–93.4%	^144^
DMIM-MSPD	Miconazole	HPLC	0.25–25 μg g^–1^	0.033 μg g^–1^	Fish	94.4–97.3%	^145^

a DCE = alpha-(2,4-dichlorophenyl)-1*H*-imidazole-1-ethanol; DMIMs = dummy molecularly imprinted microspheres; DMIP = dummy molecularly imprinted polymer; DSPE = dispersive solid phase extraction; MSPD = matrix solid-phase dispersion extraction; PMIPS = photoresponsive surface molecularly imprinted polymer shell; PS-*co*-PMMA = poly­(styrene-*co*-methacrylic acid); PT-μ-SPE = pipet tip-based on microsolid phase extraction; SMIPs = surface molecularly imprinted polymers; SPE = solid phase extraction.

Manzoor et al.^92^ suggested a straightforward and well-thought-out method for creating a MIP that can extract and detect fluconazole from a complex matrix. Using a noncovalent method, the MIP was effectively made from methacrylic acid, ethylene glycoldimethacrylate, and acetonitrile while fluconazole served as the template molecule. UPLC–MS was used to quantify fluconazole, and the results showed a LOD ≤ 1.63 × 10^–10^ mM. Additionally, a remarkable recovery rate of 91 ± 10% was achieved. Comparing the MIP to its structural counterparts, miconazole, tioconazole, and secnidazole, revealed that it was capable of selectively recognizing fluconazole, with percentage recoveries of 51, 35, and 32%, respectively. Employing pickering emulsion polymerization, Zhang et al.^145^ created dummy molecularly imprinted microspheres that were utilized to identify azole fungicides in fish specimens (Figure 8). The fragment dummy template utilized for the imprinting process included alpha-(2,4-dichlorophenyl)-1*H*-imidazole-1-ethanol, which facilitated the selective recognition of climbazole (CBZ), clotrimazole (CMZ), and miconazole (MNZ). The structural characteristics of the microspheres were examined by using scanning electron microscopy. Fish specimens spiked at 0.5, 2.5, and 12.5 μg g^–1^ showed high recoveries (89.2–101.5%) and repeatability using the improved MSPD technique. For CBZ, CMZ, and MNZ, the corresponding LODs were 0.045, 0.036, and 0.033 μg g^–1^. The method’s ease of use, speed, and minimal solvent consumption make it highly promising for quickly pretreating fish samples with azole fungicides. To detect trace griseofulvin in milk, Yang et al.^146^ created a novel photoresponsive surface molecularly imprinted polymer shell (PMIPS). The PS-*co*-PMAA@PMIP was synthesized through precipitation polymerization, utilizing griseofulvin as the template molecule, PS-*co*-PMAA as the base material, an azobenzene derivative as the functional monomer, and triethanolamine trimethacrylate as the cross-linking agent (Figure 9). The PMIPS demonstrated reversible photocontrolled incorporation and release of griseofulvin, as well as high selectivity toward it compared to its structural analogues. There were two linear correlations found between the concentrations of griseofulvin (0 to 6.4 × 10^–6^ M and 6.4 × 10^–6^ to 2.8 × 10^–5^ M). The PMIPS demonstrated a high degree of selectivity for griseofulvin in comparison to its structural analogues, alongside a reversible mechanism for the photocontrolled uptake and release of griseofulvin. Furthermore, the PMIPS proved to be effective in detecting griseofulvin within complex matrices

**Figure 8:**
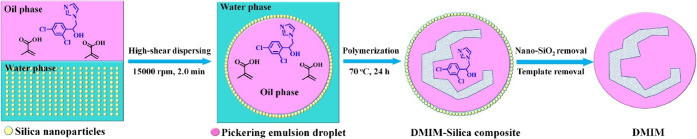
A diagrammatic illustration of the process of dummy imprinting through Pickering emulsion polymerization. Reproduced from ref 145 with permission from Elsevier.

**Figure 9:**
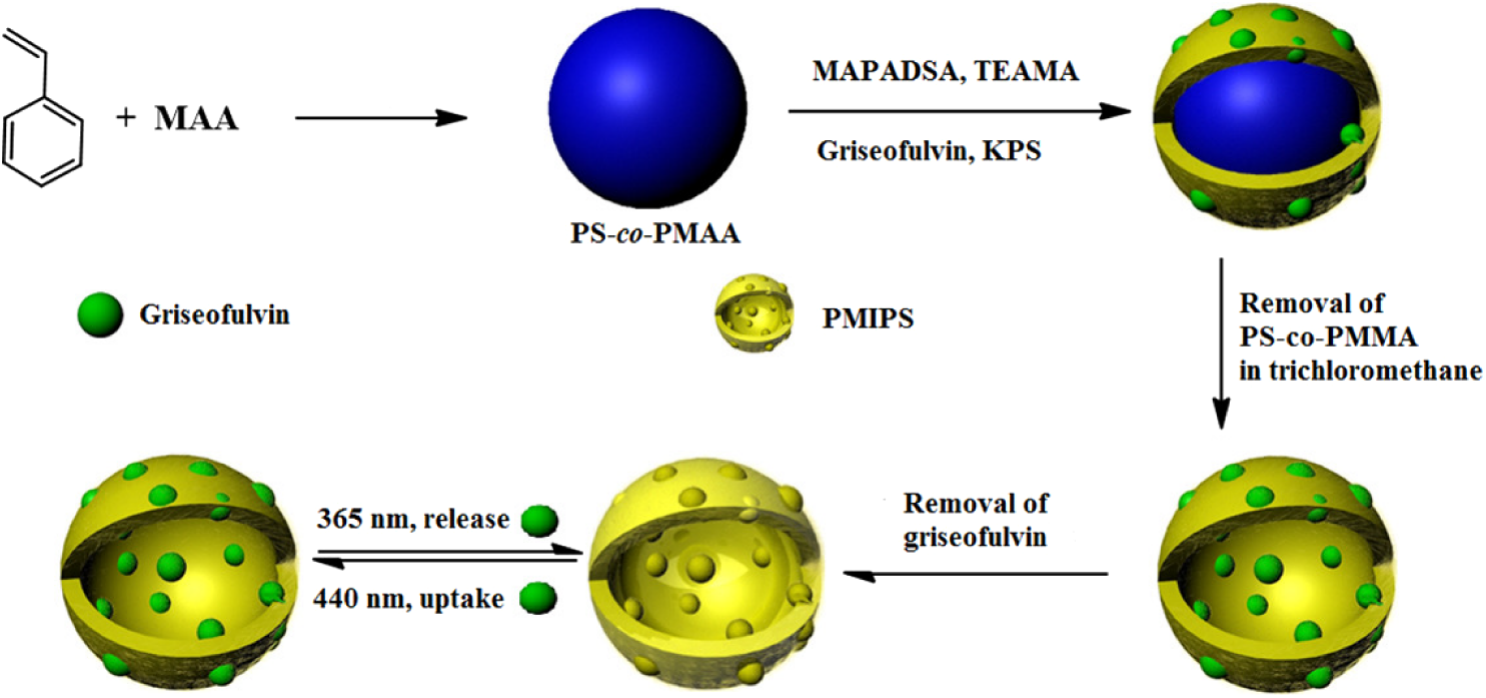
Synthetic pathway for the development of a photoresponsive molecularly imprinted polymer shell. Reproduced from ref 146 with permission from Elsevier.

### 5.3 Antiviral Drugs

The approaches to addressing viral diseases primarily involve prevention through vaccination and treatment via antiviral medications. Over 220 distinct viruses have been identified as capable of infecting humans. In situations in which an effective vaccine is not available, the presence of safe and efficacious treatments becomes essential, particularly during the initial phases of a severe viral outbreak. Currently, there are clinically approved antiviral drugs available for only 10 specific viruses, despite the existence of over 220 viruses capable of infecting humans.^150^ Table 3 displays the use of MIPs in the examination of antiviral medications that include halogens.

**Table 3:** Determination of Halogen-Containing Antiviral Drug Molecules Employing Different Types of MIP-Based Electrochemical Sensors^a^

Method	Analyte	Technique	Linear Range	LOD	Sample	Recovery	ref.
MIP-SPE	Efavirenz	LC-PDA	0.2–1.0 mg/L	0.41 μg/L	Wastewater	97%	^151^
MIP-HPLC	Efavirenz	HPLC–UV	50–300 μg/L	17.3 μg/L	Serum and urine	82.2–95.2%	^89^
MIP-DSPE	Efavirenz	HPLC-PDA	-	1.06 μg L^–1^	Water	97.20–99.68%	^152^
PT–SPE–HM–MIP	Efavirenz	HPLC–UV	0.25 to 10 μg/mL	0.23 μg/mL	Plasma	83.72%	^153^
PGE/MIP	Sofosbuvir	DPV	1.0 × 10^–11^–1.0 × 10^–13^ M	3.1 × 10^–14^ M	Tablet and plasma	97.43 and 101.14%	^154^
MIP-(fMWCNTs&RGO)-GCE	Sofosbuvir	DPV	0.53–74.13 ng/mL	0.05 ng/mL	Tablet and urine	93.34–102.68%	^90^
MIP-AuNPs/N, S@GQDs/PGE	Sofosbuvir	DPV	1–400 nM	0.36 nM	Tablet and plasma	98.4–104.5%	^155^
UMI@B_3_N_3_/BuMA@MIP/GCE	Umifenovir	EIS, DPV	0.50–7.50 pM	48.20 fM	Serum and urine	98.87–102.45%	^105^
PCD-MIP/PGE	Favipiravir	DPV	5 × 10^–^6–1 × 10^–3^ mol/L	1.67 μmol/L	Tablet and plasma	95.28–113.84%	^156^
MIP-Co/Ni@MOF	Favipiravir	DPV	0.1–151 nM	0.075 nM	Water, urine, and plasma	95.5–109.9%	^106^
MoS_2_@MIP core–shell nanocomposite	Favipiravir	DPV	0.01–100 nM	0.002 nM	Urine and plasma	81.6–119.0%	^157^
MIP/AuNPs/NiS_2_ NS/BC/GCE	Favipiravir	DPV	0.42–1100 nM	0.13 nM	Tablet and serum	99.2–102.1%	^104^
Pd/Co–Mn-MOF-74/GCE	Favipiravir	SWV	5–25 μM	0.003 μM	Urine and plasma	96–102.8%	^159^

a AuNPs = gold nanoparticles; B3N3 = borazine; BC = biomass-derived carbon; BuMA = butyl methacrylate monomer; Co/Ni@MOF = Co/Ni metal–organic-framework; DSPE = dispersive solid-phase extraction; f-MWCNTs = functionalized multiwalled carbon nanotubes; GCE = glassy carbon electrode; HM–MIP = hollow mesoporous molecularly imprinted polymer; MoS2 = molybdenum disulfide; N,S@GQDs = N,S codoped graphene quantum dots; NiS2 NS = nickel disulfide nanospheres; PCD = poly carbidopa; Pd/Co–Mn-MOF-74 = palladium supported on mixed-metal–organic framework; PGE = pencil graphite electrode; PT–SPE = pipet-tip solid-phase extraction; RGO = reduced graphene oxide; SPE = solid-phase extraction; UMI = umifenovir.

A straightforward approach for evaluating the level of efavirenz using MIP nanoparticles was developed by Pourfarzib et al.^89^ In particular, a dispersive solid-phase extraction was used for removing efavirenz from serum and urine, followed by HPLC–UV analysis. Efavirenz was used as a template molecule and methacrylic acid as a functional monomer in the mini emulsion polymerization process to create the imprinted nanoparticles. When the MIPs’ molecular recognition capabilities, binding ability, and selectivity were assessed, the findings showed that the produced MIPs exhibited a high level of unique binding for efavirenz in aqueous solutions. The extraction process, when combined with HPLC analysis, produced a linear calibration curve within the concentration range of 50–300 μg/L, with the highest recovery rates of 95.2% for serum samples and 92.7% for urine samples. Tawab et al.^90^ constructed and refined a biomimetic electrochemical sensor to detect sofosbuvir in urine samples, pharmaceutical formulations, and pure forms (Figure 10). The characterization of this electrochemical sensor involved techniques such as cyclic voltammetry, electrochemical impedance spectroscopy, and atomic force microscopy. Differential pulse voltammetry was utilized to establish the calibration curve and optimize the critical factors influencing the sensor performance. The sensor demonstrated a linear detection range from 0.53 to 74.13 ng/mL, with a notable detection limit of 0.05 ng/mL achieved through DPV, alongside commendable reproducibility and selectivity. Furthermore, the imprinted sensor was effectively employed to analyze sofosbuvir across pure samples, urine, and two distinct pharmaceutical preparations, while also conducting an electrochemical stability assessment. A surface-imprinted electrochemical sensor with borazine (B3N3) assistance was created by Cetinkaya et al.^105^ to detect Umifenovir (UMI) in urine and serum samples (Figure 11). The sensor was created through a photopolymerization process utilizing butyl methacrylate (BuMA) as the functional monomer to coordinate with the template molecules. To assess the electrochemical characteristics throughout the various stages of MIP fabrication, cyclic voltammetry and electrochemical impedance spectroscopy were employed. Under optimized experimental conditions, the sensor demonstrated a linear range of 0.50–7.50 pM for DPV and 0.25–5.00 pM for EIS. Furthermore, the sensor displayed a strong ability to selectively recognize UMI molecules, even in the presence of structural analogues and other potential interferents.

**Figure 10:**
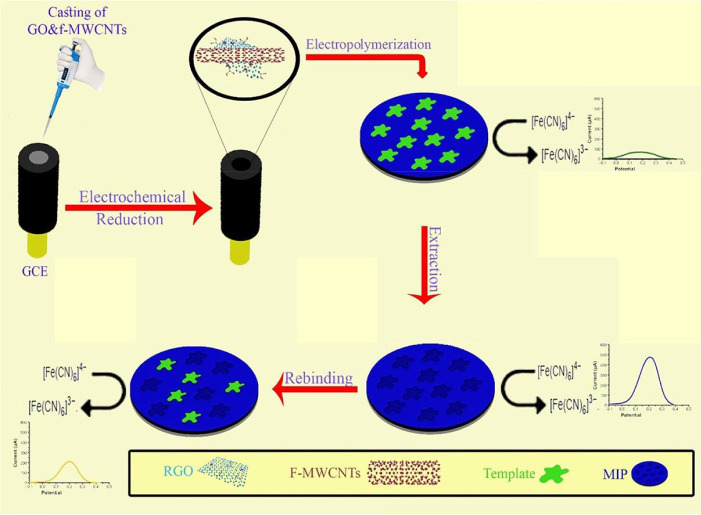
A diagrammatic illustration depicting the process involved in the fabrication of MIP-(fMWCNTs and RGO)-GCE. Reproduced from ref.^90^ with permission from Elsevier.

**Figure 11:**
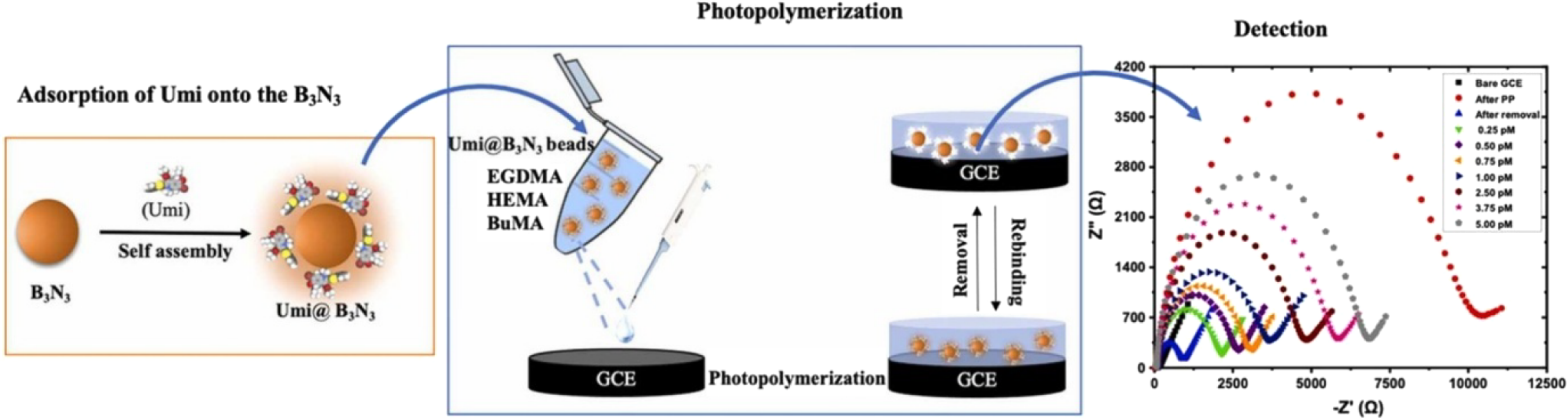
Schematic illustration of the UMI@B3N3/BuMA@MIP/GCE preparation. Reproduced from ref.^105^ with permission from Elsevier.

### 5.4 Antibacterial Drugs

Antibiotics serve the purpose of treating or preventing bacterial infections by eliminating bacteria and inhibiting their reproduction. Recently, there has been a growing focus on developing contemporary, highly accurate, and rapid techniques for identifying antibiotic compounds and their metabolites. Identifying the medications may contribute to improved results and the development of a more effective anti-infection treatment strategy.^160^ Various methodologies for analyzing halogenated antibacterial agents using MIP techniques are detailed in Table 4.

**Table 4:** Determination of Halogen-Containing Antibacterial Drug Molecules Employing Different Types of Mip-Based Electrochemical Sensors^a^

Method	Analyte	Technique	Linear Range	LOD	Sample	Recovery	ref
MIP/Au/PEDOT/GCE	Moxifloxacin	DPV	0.004–20 μM	1.109 nM	milk and honey	98.18–100.72%	^161^
MISPE	Moxifloxacin	UPLC-MS/MS	0.2–1.2 μg/mL	0.032 μg/mL	Tablets and urine	92.77–111.38%	^162^
MIP-MOX	Moxifloxacin	Potentiometric	1.0 × 10^–5^- 1.0 × 10^–2^ M	1.7 × 10^–6^ M	Tablets and urine	96.6–102.8%	^163^
CdTe@SiO_2_@MIP	Chloramphenicol	Fluorescence	40–500 μg L^–1^	5.0 μg L^–1^	Serum and milk	95.4–105.3	^164^
MIPs/BiOBr/ITO	Chloramphenicol	PEC	0.01–1000 ng mL^–1^	3.02 pg mL^–1^	Water	94.0–106.1%	^109^
MISPE-HPLC	Chloramphenicol	HPLC-MS/MS	0.1–2 ng·mL^–1^	0.02 μg L^–1^	Milk	96.04–108.68%	^165^
MIPs@SiO_2_–FITC	Ciprofloxacin	Fluorescence	4.04–250 nM	4.04 nM	Water	83.87–105.17%	^166^
MIP-ECL	Ciprofloxacin	ECL	2 × 10^–12^- 3 × 10^–9^ M	5.98 × 10^–13^ M	Animal	92–111%	^167^
MIP-Fluorescence	Ciprofloxacin	Fluorescence	0.5–100 μg L^–1^	92 ng L^–1^	Food	85.4–86.6%	^168^
MIP-dual/MWCNT-ZIF8/GCE	Gatifloxacin	DPV	1 × 10^–14^-1 × 10^–7^ M	2.61 × 10^–15^ M	Water	96.5–105%	^169^
GTFX-MIPCH	Gatifloxacin	DRM	10^–12^–10^–6^ M	1 × 10^–14^ M	Water	85.1–102%	^170^
MIP-SPME	Norfloxacin	HPLC	-	0.15 μg L^–1^	Marine	90.1–102.7%	^171^
MIP-CdTe QDs	Norfloxacin	Fluorescence	0.5–28 μM	0.18 μM	Water	96.2–106.0%	^172^
MIPs@BiOI/ITO	Norfloxacin	PEC	0.1–1000 nM	0.04 nM	Water	94.7–113%	^173^
MIPs@TiO_2_–C/ITO PEC	Ofloxacin	PEC	0.01–3000 ng mL^–1^	2.91 ng mL^–1^	Water	96.6–105.8%	^174^
CDs/Eu^3+^@MIP	Ofloxacin	Fluorescence	0.83–40 nM	0.25 nM	-	-	^175^
MIP-RECS	Ofloxacin	DPV	0.1–100 μM	13.2 nM	Milk	97.6–104.8%	^176^
MIP/sol–gel/MWCNT/Au	Clindamycin	DPV, SWV	5.0 × 10^–7^ – 8.0 × 10^–5^ M	2.44 × 10^–8^ M	Urine	93.2–101.9%	^177^
MISPE-DLLME-HPLC	Enoxacin	HPLC	0.05–10 μg L^–1^	0.012 μg L^–1^	Water	89.67–100.5%	^178^
MUCPs@MIP	Enoxacin	Fluorescence	3.90 × 10^–3^–0.25 μm	1.12 × 10^–6^ μm	Fish	101.7–104.13%	^179^
MIP-HPLC	Dicloxacillin	HPLC–DAD	15–2500 μg L^–1^	1.9 μg kg^–1^	Milk	94–99	^180^
AuNPs/RGO/SWCNT/GCE	Pefloxacin	DPV	5.0 × 10^–7^ – 2.0 × 10^–5^ M	1.6 × 10^–8^ M	Milk	87–92%	^181^
MIP/BPNS-AuNPs	Pefloxacin	LSV	0.005–10 μM	0.08 nM	Milk, and orange juice	95.33–102.6%	^182^
BSA@MIPs	Pefloxacin	HPLC	1–100 μg/L	1.31 μg/L	Egg	89.5–98.6	^183^
MIPDA/peptide/GNPs/GCE	Vancomycin	EIS	10 pM–100 μM	10 pM	Calf serum and food	92.16–104.67%	^184^
SMISPE-LC–MS/MS	Vancomycin	LC–MS/MS	1–100 ng/mL	0.5 ng/mL	Mice plasma	94.3–104.0%	^185^
Alginate@TiO_2_/MIP-GCE	Vancomycin	DPV	10–100 pM	2.808 pM	Water and serum	100.79–101.6%	^186^
MIP-SPME/LC–MS	Linezolid	LC–MS	1–50 μg/mL	0.198 ng/mL	Serum	99.29–103.29%	^187^
MIPSB	Cefaclor	HPLC	20–320 ng mL^–1^	3.5 ng mL^–1^	Water	87.1–98.6%	^188^
PANI-GOx-MIP-QDs	Lomefloxacin	Fluorescence	0.10–50.0 μg L^–1^	0.07 μg L^–1^	Food	81.5–99.6%	^189^
MIP/Fe-PC/Au	Lomefloxacin	DPV	1 to 120 nM	0.2 nM	Water and milk	86.6–105.0%	^190^
MISPE-DLLME-HPLC	Lomefloxacin	HPLC	0.05–10 μg L^–1^	0.015 μg L^–1^	Water	89.67–100.5%	^178^
MIP-SPE-HPLC	Levofloxacin	HPLC	0.01–1.50 mg/L	0.003 mg/L	Water	83.67–101.33%	^191^
LVFX-MIPCH	Levofloxacin	DRM	10^–10^ to 10^–4^ M	10^–12^ M	Water	93.33–114.81%	^192^
PGE/Au-NPs/poly(*o*PD-*co*-l-Dopa)	Levofloxacin	DPV	1.0 × 10^–6^–1.0 × 10^–2^ M	4.62 × 10^–7^ M	Tablets and plasma	90.37–105.63%	^193^
MISPE-HPLC	Cloxacillin	HPLC	0.05–1.5 μg L^–1^	0.98 μg L^–1^	Water	83.1–96.9%	^194^
RGO/AuNPs-SPCE	Cloxacillin	DPV	110–750 nM	36 nM	Milk	98.6–101.8%	^195^
MIM-HPLC	Cloxacillin	HPLC	0.5–500 μg g^–1^	0.03 μg g^–1^	Shrimp	80.9–94.9%	^196^
MMIP-NPs	Gemifloxacin	Potentiometry	1 × 10^–3^ – 1 × 10^–10^ M	6.4 × 10^–11^ M	Tablets and plasma	99.21%	^197^
MISPE	Gemifloxacin	UPLC–MS/MS	0.1–1.6 μg mL^–1^	4 ng mL^–1^	Tablets and urine	90.24–112.8%	^198^
MIMSM	Sparfloxacin	Fluorescence	0.05–2.0 μg/mL	0.012 μg/mL	Serum	88.8–102%	^199^
N-GQDs-MIP-ZnO/CF-Fe_3_O_4_	Sparfloxacin	Fluorescence	0.10–100.0 μg L^–1^	0.10 μg L^–1^	Milk	93.0–100.8%	^200^
N-GQD@ZnO@MIP, CdTe QD@ZnO@MIP	Sparfloxacin	Fluorescence	0.10 to 100.0 μg L^–1^	0.10 μg L^–1^	Food	93.3–103.4%	^201^
MISPE-UPLC-MS/MS	Teicoplanin	UPLC-MS/MS	1.0–20.0 μg/L	0.05 μg/L	Milk and biological	75.2–91.4%	^202^
SMIP-dSPE	Teicoplanin	HPLC	5–100 μg L^–1^	5 μg L^–1^	Water	81.4–94.6%	^203^
QDs@APBA@MIPs	Teicoplanin	Fluorescence	1.0–17 μM	0.714 μM	Urine and food	91.83–106.67%	^204^
MIP/MnO-Fe_3_O_4_@C/GCE	Thiamphenicol	DPV	0.01–40 μM	0.007 μM	Food	98.03–108.43%	^205^
N,S-CDs@MIPs	Thiamphenicol	Fluorescence	10–300 μg/L	6.20 μg/L	Water and food	92.08–108.4%	^206^
MIP-PEC	Thiamphenicol	PEC	1.0 × 10^–9^- 3.5 × 10^–6^ M	5.0 × 10^–10^ M	Food	90–97%.	^207^
N-CDs@MIPs	Chlortetracycline	Fluorescence	6.67–111.33 μM	3.19 μM	Milk	96.7–109.8%	^208^
MIP-PEC	Chlortetracycline	PEC	0.5 nM- 10 μM	0.17 nM	Water and food	86.4–112.0%	^209^
TiO_2_@Ti_3_C_2_T_*x*_/MIP	Chlortetracycline	DPV	0.06–1000 nM	0.027 nM	Food	97.9–102.4%	^210^
CD@FF-MIP	Florfenicol	Fluorescence	3–50 μM	-	Milk	95.8–98.2%	^211^
RAM–MMIPs	Florfenicol	HPLC	-	10.5 μg/L	Bovine serum	95.6–102.1%	^212^
MIP–PEC	Florfenicol	PEC	1.0 × 10^–4^–1.0 × 10 ^4^ ng mL^–1^	6.4 × 10^–5^ ng mL^–1^	Food	84.0–111%	^213^
OH-HNTs@*N*-GQDs@Fe_3_O_4_@MIP	Marbofloxacin	Fluorescence	0.10–25.0 μg L^–1^	0.03 μg L^–1^	Milk	94.2–99.6%	^214^
MIP-capacitive	Pazufloxacin	Capacitance	5–5000 ng·mL^–1^	1.8 ng·mL^–1^	Urine	94.0–102.0%	^215^
MISPE	Nadifloxacin	UPLC	0.1–100 μg mL^–1^	100 ng mL^–1^	Plasma	96.00%	^216^
RAM-MIPs	Sarafloxacin	HPLC	4–300 ng g^–1^	4.23 ng g^–1^	Egg	94.0–101.3%	^217^
PDA@MIP@QDs	Sarafloxacin	Fluorescence	0.10–15.0 μg L^–1^	0.05 μg L^–1^	Chicken meat	82.8 to 99.1%	^218^
MISPE–HPLC	Difloxacin	HPLC	0.01–2.0 mg/L	1.07 μg/L	Pork	78.16–87.92%	^219^
MIP-PMME	Difloxacin	HPLC	5.0–200 ng/mL	0.8 ng/mL	Milk	92.4–98.2%	^220^
MISPE-DLLME-HPLC	Fleroxacin	HPLC	0.05–10 μg L^–1^	0.013 μg L^–1^	Water	89.67–100.5%	^178^
MUCPs@MIP	Fleroxacin	Fluorescence	1.69 × 10^–3^–0.22 μM	4.06 × 10^–7^ μM	Fish	90.1–105.83	^179^
MIP-ELISA	Flumequine	ELISA	2.18 × 10^–3^ to 9.08 μg/mL	4.0 μg/L	Food	80.7–90.7%	^221^
MISPE–HPLC	Flumequine	HPLC	0.020–2.0 mg/L	1.10 μg/L	Pork	81.38–93.5%	^219^
MPCMIP	Demeclocycline	HPLC	0.981–50.7 μg/mL	0.215 μg/mL	Water and food	98.1–109%	^222^
MWCNTs/MIPs	Enrofloxacin	DPV	2.8 pM–28 μM	0.9 pM	Marine	96.4%–102%	^223^
MIP-SPE	Enrofloxacin	SWV	0.01–0.1 mM	0.02 mM	Standard solution	-	^224^
MIP-ECL	Enrofloxacin	ECL	0.1 nM–1 μM	27 pM	Water	88.20–105.0%	^225^
MWCNT-COOH-RGO/MIP	Enrofloxacin	SWV	1.0 × 10^–10^ −5.0 × 10^–5^ M	2.5 × 10^–11^ M	Egg	71.3–124.6%	^226^

a 3-APBA = 3-Aminophenylboronic acid; Au/PEDOT = gold/Poly­(3,4-ethylenedioxythiophene); AuNPs/RGO = gold nanoparticles/reduced graphene oxide; BiOBr = bismuthyl bromide; BiOI = bismuth oxyiodide; BPNS = black phosphorus nanocomposites; BSA = bovine serum albumin; CD = N–S codoped carbon dot; CDs/Eu3+ = carbon dots and Eu3+; CdTe QDs = cadmium telluride quantum dots; CdTe@SiO2 = silica nanospheres embedded CdTe quantum dots; DLLME = dispersive liquid–liquid microextraction; DRM = Debye diffraction ring measurement; dSPE = dispersive solid-phase extraction; ECL = electrochemiluminescence; Fe3O4 = magnetic nanoparticles; Fe-PC = Fe-doped porous carbon; FF = florfenicol; FITC = fluorescein isothiocyanate; GNPs = gold nanoparticles; Gox = graphene oxide; GTFX-MIPCH = gatifloxacin molecularly imprinted two-dimensional photonic crystal hydrogel; ITO = indium tin oxide; LVFX = levofloxacin; MIMSM = molecularly imprinted mesoporous silica microspheres; MIPCH = molecularly imprinted photonic crystal hydrogels; MIPDA = molecularly imprinted polydopamine; MIPSBE = molecularly imprinted polymeric stir bars-based extraction; MISPE = molecularly imprinted solid phase extraction; MMIP = magnetic molecularly imprinted polymer; MnO-Fe3O4@C = MnO-Fe3O4 composite nanospheres coated with a carbon layer; MOX = moxifloxacin; MPCMIP = magnetic porous cellulose molecularly imprinted polymer; MWCNT = multiwalled carbon nanotube; N-GQDs = nitrogen-doped graphene quantum dots; OH-HNTs = hydroxylated-halloysite nanotubes; oPD = o-phenylenediamine; PANI = polyaniline; PDA = polydopamine; PEC = photoelectrochemical; PMME = polymer monolith microextraction; RAM = restricted access media; RECS = ratiometric electrochemical sensor; SMIP = surface molecularly imprinted polymer; SMISPE = surface molecularly imprinted solid-phase extraction; SPE = screen-printed gold electrodes; SPME = solid-phase microextraction; SWCNT = single-walled carbon nanotubes; Ti3C2Tx = titanium carbide; TiO2-C = COOH functionalized TiO2; ZIF8 = zeolitic imidazolate framework 8.

Using 3D flower-like BiOBr with strong photocurrent sensitivity activity and MIPs with many recognition features, Zhang et al.^109^ created a photoelectrochemical sensor (Figure 12). The sensor’s selectivity to CAP was enhanced during the PEC sensing process by preparing MIPs using a straightforward thermal polymerization procedure, which produced many recognition sites. With a low detection limit of 3.02 pg mL^–1^, the results demonstrated that the photocurrent response signal produced by MIPs/BrOBr/ITO sensor was directly proportional to the logarithm of CAP quantity across the range of 1.00 × 10^–2^ to 1.00 × 10^3^ ng mL^–1^. In addition to being inexpensive and useful for determining CAP in actual samples, the MIPs-PEC sensor demonstrated excellent specificity and durability. Furthermore, the sensor exhibited promising practicality for detecting CAP in actual water samples, suggesting its potential as a viable alternative for environmental monitoring of water quality. A novel electrochemical sensor with molecular imprinting was developed by Huang et al.^169^ for the specific detection of gatifloxacin, utilizing dual functional monomers. As dual functional monomers, *p*-aminobenzoic acid (p-ABA) and nicotinamide (NA), were employed in the electropolymerization of MIP, with GTX serving as the template component (Figure 13). The sensor has a low detection limit of 2.61 × 10^–15^ M and a broad linear detection range of 1.00 × 10^–14^ to 1.00 × 10^–7^ M. The method exhibited commendable recovery rates between 96.5% and 105%, with relative standard deviations ranging from 2.4% to 3.7% in actual water samples, highlighting its efficacy for detecting antibiotic contaminants. A photoelectrochemical sensor enhanced by MIP was constructed by Zhang et al.^174^ for the highly sensitive detection of ofloxacin. The detection limit was determined to be 2.91 ng mL^–1^, and under ideal circumstances, the MIPs@TiO_2_–C/ITO PEC sensor showed a broad linear correlation with the concentration of ofloxacin, spanning from 0.01 to 3000 ng mL^–1^. In actual water samples, the constructed MIPs-PEC sensor demonstrated outstanding stability, resistance to interference, and feasibility. According to these findings, a potential technique for keeping an eye on antibiotics and other dangerous compounds in the environment was offered by combining PEC and MIPs. Li et al.^182^ constructed a very robust electrochemical sensing platform for precisely determining pefloxacin (PEF) utilizing MIP technology. The incorporation of gold nanoparticle/black phosphorus nanocomposites (BPNS-AuNPs) significantly improves the stability and electrochemical performance of black phosphorus while also providing an expanded surface area that facilitates the creation of additional imprinted sites for the selective binding of PEF. MIP/BPNS-AuNPs have a high sensitivity (3.199 μA μM^–1^), very low-level detection (0.80 nM), and a wide linear detection region (0.005–10 μM). The strong binding affinity of MIP/BPNS-AuNPs for PEF is demonstrated, even when structural analogues are present. When it comes to detecting PEF in actual milk and orange juice samples, the MIP sensor consistently shows good sensitivity. For at least 5 weeks, the MIP/BPNS-AuNPs/GCE sensor ensures steady and reliable voltammetric functioning, which is crucial in real-world applications.

**Figure 12:**
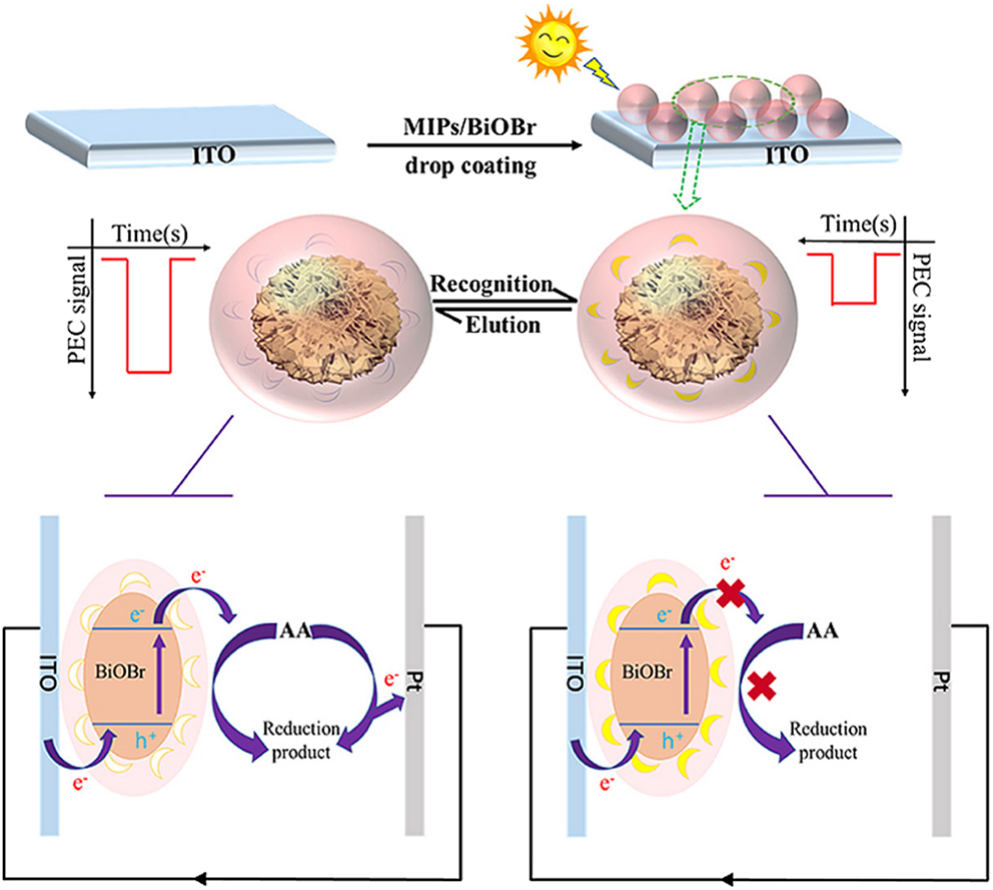
Schematic representation elucidates the operational principle of the MIPs/BiOBr/ITO photoelectrochemical sensor designed to detect CAP. Reproduced from ref 109 with permission from Elsevier.

**Figure 13:**
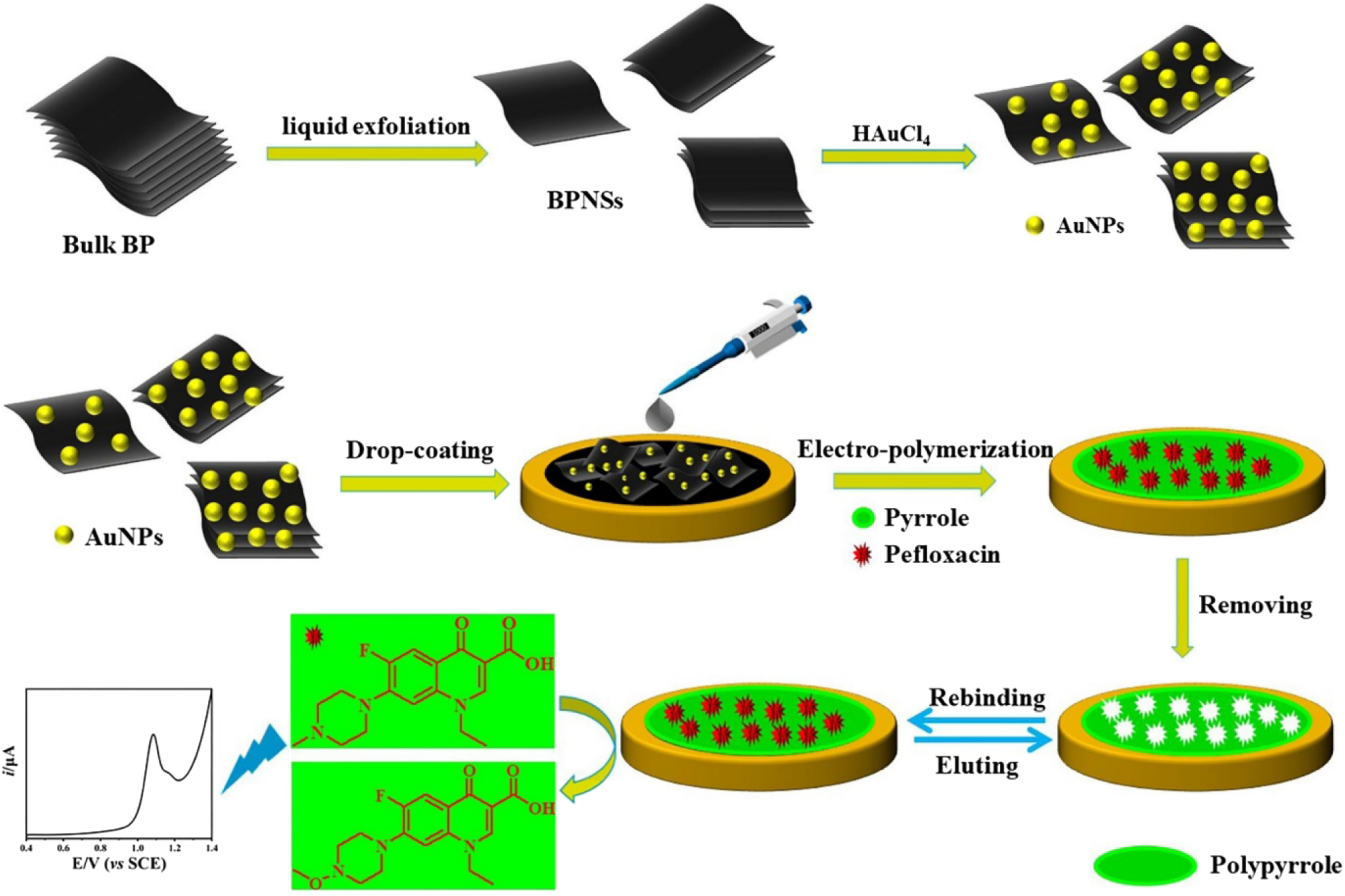
Diagrammatic representation of the MIP/BPNS-AuNPs/GCE preparation. Reproduced from ref 182 with permission from Elsevier.

## 6 Conclusion

This is a detailed review of the status, applications, and current literature data of MIPs for the detection of antiparasitic, antifungal, and antiviral drug molecules. It is the first study to review the current developments on this subject, as it covers halogenated drug molecules. Considering the advancing technology and the health problems that come with it, the strategy of MIPs to provide an economical and portable detection solution for on-site investigations in different areas has been put forward. The basic working principles, manufacturing techniques, materials, and methodologies related to MIPs are given by comparison with other source data. Determination of halogen-containing antiparasitic, antifungal, and antiviral drug molecules using different types of MIP-based electrochemical sensors has been carried out in different environments such as standard solution, water, vegetables, serum, urine, food, fruit, honey, tablet, capsule, pharmaceutical/biological plasma, milk, chicken meat, fish, and wastewater. Driven by the demand for environmentally friendly, affordable, and reliable analytical techniques, molecularly imprinted polymers have received increasing attention from various research groups, especially in the past decade. This interest is particularly evident in the analysis of active pharmaceutical compounds from pharmaceutical formulations, biological fluids, and environmental materials. MIP structures used in the quantitative analysis of halogen-containing active pharmaceutical ingredients are synthesized and characterized based on the principle of “environmentally friendly materials” while incorporating “green chemistry” strategies. In their current forms, they are applicable in various fields. Paul Anastas defines “green chemistry” or “green approach” as “the creation of chemical products and processes that are more environmentally friendly”, aiming to minimize negative health and environmental impacts. In scientific terms, some principles of green chemistry originate from analytical chemistry, while others are mainly associated with the stages of chemical synthesis. In green analytical chemistry, incorporating nonhazardous green solvents or solvent mixtures and readily degradable reagents necessitates adopting renewable resources into the system. During the production of all pharmaceutical forms, analytical procedures evaluate the quality control of raw materials and final products. Attention is paid to the analytical validation parameters and the extent to which the analysis process affects the environment, especially in terms of waste solvent monitoring. From the past to present, all of these steps have been attempted to be managed with a sensitive approach in every sector. Analysis studies conducted in the context of green analytical chemistry thoroughly elaborate on the principles of this sustainable approach. Additionally, metric systems have been established^227^ to provide a clear answer to the question, “Green or not?”. The metric measurement suggestions encompass several indicators to obtain insights into diverse aspects of a current issue. Moreover, several computational programs have been created following the green chemistry protocol to assess a process’s environmental sustainability. Although numerous studies emphasizing the environmental friendliness of MIPs were cited in this review article, no explanation was provided because there was no statement “emphasizing this issue” in any research article. As the importance of environmentally sustainable MIPs in green chemistry increases, along with the urgent demand for greenness metrics, we anticipate that their production will become essential.

## 7 Future Perspectives

In the final part of this review, we identified about two hundred studies on the determination of halogenated drugs from our literature search in the “Web of Science (all fields)” database. When we performed an exact search with the keyword “molecularly imprinted polymer”, we discovered approximately 17,000 studies. Approximately 5000 studies focused on quantitative analysis, with about one-fifth concentrating on pharmaceuticals. Factors such as a growing and aging population, advancements in healthcare services and medication access, increased average life expectancies, and rising welfare and awareness will undoubtedly enhance both drug consumption and its scrutiny in the future. Recently, there has been significant focus on developing MIPs as specialized materials for a variety of applications.

Molecularly imprinted polymer technology offers considerable advantages in attaining low detection limits and fostering environmentally sustainable methods for analyzing pharmaceutical formulations, biological fluids, and environmental samples. Molecularly Imprinted Polymers (MIPs) are dependable, environmentally friendly sensor technologies that aid nanomedicine, pharmaceutical formulations, food quality assessment, and environmental management. We believe that it highlights the potential to improve the quality of life in nature through sustainable practices and technological advancements. As exciting research in this field progresses, commercial development of molecular imprinting sensors remains in its early stages. Detection methods utilizing MIPs are continually evolving and finding applications across various fields. Therefore, information about commercialization and patents in this area should be easily accessible. The affordability, exceptional stability, and consistently improving performance of MIPs position these polymers as premier synthetic materials for molecular recognition across various scientific disciplines. Additionally, other aspects of MIP development and MIP-based sensors can be improved. Increasing the variety of functional monomers and conducting thorough studies of their synthesis for the template molecule will significantly improve the design and selectivity of MIPs. When preparing sensors, removing the template molecule, and reattaching them, it is essential to investigate options to reduce the attachment time and increase the sensor’s stability. In this way, the sensor can be reused, saving time by eliminating the need to reread the entire preparation process.

## References

[1] Sandle, T. Antibiotics and Preservatives. In Pharmaceutical Microbiology; Elsevier, 2016. pp. 171–183. 10.1016/B978-0-08-100022-9.00014-1.

[2] Pai, M. P. ; Cottrell, M. L. ; Kashuba, A. D. M. ; Bertino, J. S., Jr. Pharmacokinetics and Pharmacodynamics of Anti-Infective Agents. In Mandell, Douglas, and Bennett’s Principles and Practice of Infectious Diseases; Elsevier, 2015. Vol. 1, pp. 252–262.e2. 10.1016/B978-1-4557-4801-3.00019-9.

[3] Finch R. G. (2010). Principles
of Anti-Infective Therapy. Infect. Dis..

[4] Finberg, R. W. ; Guharoy, R. Clinical Use of Anti-Infective Agents; Springer International Publishing: Cham, 2021. 10.1007/978-3-030-67459-5.

[5] Faleye O. S., Boya B. R., Lee J.-H., Choi I., Lee J. (2024). Halogenated
Antimicrobial Agents to Combat Drug-Resistant Pathogens. Pharmacol. Rev..

[6] Cavallo G., Metrangolo P., Milani R., Pilati T., Priimagi A., Resnati G., Terraneo G. (2016). The Halogen Bond. Chem. Rev..

[7] Turunen L., Erdélyi M. (2020). Halogen Bonds of Halonium Ions. Chem. Soc. Rev..

[8] Zhu Z., Wang G., Xu Z., Chen Z., Wang J., Shi J., Zhu W. (2019). Halogen Bonding
in Differently Charged Complexes: Basic
Profile, Essential Interaction Terms and Intrinsic σ-Hole. Phys. Chem. Chem. Phys..

[9] Li X.-Z., Wei X., Zhang C.-J., Jin X.-L., Tang J.-J., Fan G.-J., Zhou B. (2012). Hypohalous
Acid-Mediated Halogenation of Resveratrol and Its Role
in Antioxidant and Antimicrobial Activities. Food Chem..

[10] Babii C., Mihalache G., Bahrin L. G., Neagu A.-N., Gostin I., Mihai C. T., Sârbu L.-G., Birsa L. M., Stefan M. (2018). A Novel Synthetic
Flavonoid with Potent Antibacterial Properties: In Vitro Activity
and Proposed Mode of Action. PLoS One.

[11] Boya B. R., Lee J.-H., Lee J. (2022). Antibiofilm and Antimicrobial
Activities
of Chloroindoles Against Uropathogenic Escherichia Coli. Front. Microbiol..

[12] Sayed A. M., Alhadrami H. A., El-Hawary S. S., Mohammed R., Hassan H. M., Rateb M. E., Abdelmohsen U. R., Bakeer W. (2020). Discovery of Two Brominated
Oxindole Alkaloids as Staphylococcal DNA Gyrase and Pyruvate Kinase
Inhibitors via Inverse Virtual Screening. Microorganisms.

[13] Patel P., Parmar R. (2024). Validation of UV Spectrophotometric
Method for Estimation
of Bedaquiline Fumarate in Bulk and Pharmaceutical Formulations. J. Res. Pharm..

[14] Zhao R., Bi S., Shao D., Sun X., Li X. (2020). Rapid Determination
of Marbofloxacin by Surface-Enhanced Raman Spectroscopy of Silver
Nanoparticles Modified by β-Cyclodextrin. Spectrochim. Acta, Part A.

[15] Wang X.-L., Chen S.-L., Zhao H.-C., Jin L.-P., Li X. (2005). Europium Sensitized
Chemiluminescence Determination of Pazufloxacin Mesylate in Urine
and Serum. Anal. Lett..

[16] Ling X., Deng D. W., Zhong W. Y., Yu J. S. (2008). Quantitative
Determination
of Pazufloxacin Using Water-Soluble Quantum Dots as Fluorescent Probes. Prime.

[17] Chen J., Wang C., Huang X., Wan R., Zhu Z., Sun G., Wang X., Chen H., Han L., Li L., Li H., Chi Z. (2025). Quantum Dots-Engineered
Flexible Hydrogel as Plant-Wearable
Sensor for On-Site Profiling Dynamic Pesticide Degradation. Adv. Funct. Mater..

[18] Zhou J.-W., Li P.-L., Ji P.-C., Yin K.-Y., Tan X.-J., Chen H., Xing X.-D., Jia A.-Q. (2024). Carbon Quantum Dots
Derived from Resveratrol Enhances Anti-Virulence Activity against
Pseudomonas Aeruginosa. Surf. Interfaces.

[19] Xue B., Hou A., Du Y., Qi Y., Jiang H., Zhou H., Zhou Z., Chen H. (2023). AIE Donor-Dependent
Photosensitizer
for Enhance Photodynamic Antibacterial Interface. Surf. Interfaces.

[20] Mkhize B., Court R., Castel S., Joubert A., van der
Merwe M., Wiesner L. (2024). Development and Validation of a Liquid
Chromatography Tandem Mass Spectrometry Assay for the Analysis of
Bedaquiline and M2 in Breast Milk. J. Mass Spectrom.
Adv. Clin. Lab.

[21] Maia A. S., Paíga P., Delerue-Matos C., Castro P. M. L., Tiritan M. E. (2020). Quantification
of Fluoroquinolones in Wastewaters by Liquid Chromatography-Tandem
Mass Spectrometry. Environ. Pollut..

[22] Yikilmaz Y., Filazi A. (2015). Detection of Florfenicol
Residues in Salmon Trout via
GC–MS. Food Anal. Methods.

[23] Wang M. J., Wang K., Zhang J. H., Zhu H. F., Sun K., Shen R. L. (2019). Determination and
Pharmacokinetic Study of Tedizolid
in Rat Plasma by UPLC–MS/MS. Lat. Am.
J. Pharm..

[24] Moore C. M., Sato K., Katsumata Y. (1991). High-Performance
Liquid Chromatographic
Determination of Cephalosporin Antibiotics Using 0.3 Mm I.D. Columns. J. Chromatogr. A.

[25] Maheshwaran S., Kogularasu S., Chen S.-M., Chen W.-H., Lee Y.-Y., Chang-Chien G.-P. (2023). Ultra-Trace
Detection of Sulfathiazole, an Anti-Infective
Agent and Environmental Contaminant, Using Electrochemical Sensing
with Holmium Vanadate-Graphene Oxide Nanocomposites. J. Taiwan Inst. Chem. Eng..

[26] Aftab S., Bakirhan N. K., Esim O., Shah A., Savaser A., Ozkan Y., Ozkan S. A. (2020). NH2-FMWCNT-Titanium
Dioxide Nanocomposite
Based Electrochemical Sensor for the Voltammetric Assay of Antibiotic
Drug Nadifloxacin and Its in Vitro Permeation Study. J. Electroanal. Chem..

[27] Fodey T. L., George S. E., Traynor I. M., Delahaut P., Kennedy D. G., Elliott C. T., Crooks S. R. H. (2013). Approaches for the Simultaneous Detection
of Thiamphenicol, Florfenicol and Florfenicol Amine Using Immunochemical
Techniques. J. Immunol. Methods.

[28] Kong S., Liao M., Gu Y., Li N., Wu P., Zhang T., He H. (2016). Colorimetric Recognition of Pazufloxacin
Mesilate Based on the Aggregation of Gold Nanoparticles. Spectrochim. Acta, Part A.

[29] Nawaz N., Abu Bakar N. K., Muhammad Ekramul Mahmud H. N., Jamaludin N. S. (2021). Molecularly
Imprinted Polymers-Based DNA Biosensors. Anal.
Biochem..

[30] Hirayama K., Sakai Y., Kameoka K., Noda K., Naganawa R. (2002). Preparation
of a Sensor Device with Specific Recognition Sites for Acetaldehyde
by Molecular Imprinting Technique. Sens. Actuators,
B.

[31] UludaĞ Y., Piletsky S. A., Turner A. P. F., Cooper M. A. (2007). Piezoelectric
Sensors
Based on Molecular Imprinted Polymers for Detection of Low Molecular
Mass Analytes. FEBS J..

[32] Tan Y., Yin J., Liang C., Peng H., Nie L., Yao S. (2001). A Study of
a New TSM Bio-Mimetic Sensor Using a Molecularly Imprinted Polymer
Coating and Its Application for the Determination of Nicotine in Human
Serum and Urine. Bioelectrochemistry.

[33] Peng H., Liang C., Zhou A., Zhang Y., Xie Q., Yao S. (2000). Development of a New Atropine Sulfate Bulk Acoustic Wave Sensor Based
on a Molecularly Imprinted Electrosynthesized Copolymer of Aniline
with O-Phenylenediamine. Anal. Chim. Acta.

[34] Chassaing, C. ; Sekljic, H. Approaches towards Antiparasitic Drug Candidates for Veterinary Use. In Antiparasitic and Antibacterial Drug Discovery; Wiley, 2009, pp. 117–133. 10.1002/9783527626816.ch8.

[35] Dziduch K., Greniuk D., Wujec M. (2022). The Current Directions of Searching
for Antiparasitic Drugs. Molecules.

[36] Kappagoda S., Singh U., Blackburn B. G. (2011). Antiparasitic
Therapy. Mayo Clin. Proc..

[37] Stanley, S. L., Jr Antiparasitic Agents. In Infectious Diseases; Elsevier, 2010, Vol. 2, pp. 1490–1507. 10.1016/B978-0-323-04579-7.00150-7.

[38] DRUGBANK. Antiparasitic Agents, 2024. https://go.drugbank.com/categories/DBCAT000522. (accessed 21 December 2024).

[39] Vanreppelen G., Wuyts J., Van Dijck P., Vandecruys P. (2023). Sources of
Antifungal Drugs. J. Fungi.

[40] Houšt’ J., Spížek J., Havlíček V. (2020). Antifungal Drugs. Metabolites.

[41] Assress H. A., Selvarajan R., Nyoni H., Mamba B. B., Msagati T. A. M. (2021). Antifungal
Azoles and Azole Resistance in the Environment: Current Status and
Future Perspectives—a Review. Rev. Environ.
Sci. Biotechnol..

[42] Hui S. T., Gifford H., Rhodes J. (2024). Emerging Antifungal
Resistance in
Fungal Pathogens. Curr. Clin. Microbiol. Rep..

[43] DRUGBANK Antifungal Agents, 2024. https://go.drugbank.com/categories/DBCAT000200. (accessed 21 December 2024).

[44] Kausar S., Said Khan F., Ishaq Mujeeb Ur Rehman M., Akram M., Riaz M., Rasool G., Hamid Khan A., Saleem I., Shamim S., Malik A. (2021). A Review: Mechanism
of Action of Antiviral Drugs. Int. J. Immunopathol.
Pharmacol..

[45] Ianevski A., Ahmad S., Anunnitipat K., Oksenych V., Zusinaite E., Tenson T., Bjørås M., Kainov D. E. (2022). Seven Classes of
Antiviral Agents. Cell. Mol. Life Sci..

[46] DrugBank. Antiviral Agents, 2024. https://go.drugbank.com/categories/DBCAT000066 (accessed 21 December 2024).

[47] Muteeb G., Rehman M. T., Shahwan M., Aatif M. (2023). Origin of Antibiotics
and Antibiotic Resistance, and Their Impacts on Drug Development:
A Narrative Review. Pharmaceuticals.

[48] Hutchings M. I., Truman A. W., Wilkinson B. (2019). Antibiotics:
Past, Present and Future. Curr. Opin. Microbiol..

[49] DrugBank. Anti-Bacterial Agents, 2024. https://go.drugbank.com/categories/DBCAT000104. (Accessed 21 December 2024).

[50] Elbordiny H. S., Elonsy S. M., Daabees H. G., Belal T. S. (2022). Sustainable Quantitative
Determination of Allopurinol in Fixed Dose Combinations with Benzbromarone
and Thioctic Acid by Capillary Zone Electrophoresis and Spectrophotometry:
Validation, Greenness and Whiteness Studies. Sustainable Chem. Pharm..

[51] Vashistha V. K., Bala R., Pullabhotla R. V. S.
R. (2023). Derivatizing Agents
for Spectrophotometric and Spectrofluorimetric Determination of Pharmaceuticals:
A Review. J. TAIBAH UNIV SCI..

[52] Sikorski M., Sikorska E., Khmelinskii I. V., Gonzalez-Moreno R., Bourdelande J. L., Siemiarczuk A. (2003). Photophysics
of Lumichrome on Cellulose. J. Photochem. Photobiol.
A Chem..

[53] Yuan Y. X., Li Z. H., Jiao G. Z. (2012). Rapid and Sensitive
Competitive Fluorescence
Immunoassay for the Detection of Chlortetracycline Residues in Environment. Adv. Mat. Res..

[54] Ling X., Deng D.-W., Zhong W.-Y., Yu J.-S. (2008). Quantitative Determination
of Pazufloxacin Using Water-Soluble Quantum Dots as Fluorescent Probes. Spec. Spectral Anal..

[55] Wang M.-J., Wang K., Zhang J.-H., Zhu H.-F., Sun K., Shen R.-L. (2019). Determination and Pharmacokinetic Study of Tedizolid
in Rat Plasma by UPLC–MS/MS. Lat. Am.
J. Pharm..

[56] Kowalski P., Konieczna L., Chmielewska A., Olędzka I., Plenis A., Bieniecki M., Lamparczyk H. (2005). Comparative
Evaluation between Capillary Electrophoresis and High-Performance
Liquid Chromatography for the Analysis of Florfenicol in Plasma. J. Pharm. Biomed. Anal..

[57] Zhang Z.-L., Li J.-J., Qu L.-B., Yang R. (2008). Determination of Pazufloxacin
Mesylas by Capillary Electrophoresis with Electrochemiluminescence
Detection. Chin. J. Anal. Chem..

[58] Vílchez J. L., Navalón A., Araujo L., Prieto A. (2007). Determination of Danofloxacin
and Marbofloxacin in Milk Samples by Micellar Liquid Chromatography
with Fluorescence Detection. Anal. Lett..

[59] Yatsukawa Y.-I., Ito H., Matsuda T., Nakamura M., Watai M., Fujita K. (2011). Determination
of Residual Fluoroquinolones in Honey by Liquid Chromatography Using
Metal Chelate Affinity Chromatography. J. AOAC
Int..

[60] Patel K. G., Shah P. M., Shah P. A., Gandhi T. R. (2016). Validated High-Performance
Thin-Layer Chromatographic (HPTLC) Method for Simultaneous Determination
of Nadifloxacin, Mometasone Furoate, and Miconazole Nitrate Cream
Using Fractional Factorial Design. J. Food Drug
Anal..

[61] Wu J.-E., Chang C., Ding W.-P., He D.-P. (2008). Determination
of
Florfenicol Amine Residues in Animal Edible Tissues by an Indirect
Competitive ELISA. J. Agric. Food Chem..

[62] Zhou L., Chen G., Chen M., Lu X., Xi Y., Zhi Y. (2022). Development of a Highly Sensitive
Monoclonal Antibody-Based Indirect
Competitive Enzyme-Linked Immunosorbent Assay for the Detection of
Avilamycin in Feed. Food Addit. Contam., Part
A.

[63] Wang G., Wang B., Zhao X., Xie X., Xie K., Wang X., Zhang G., Zhang T., Liu X., Dai G. (2019). Determination of Thiamphenicol, Florfenicol and Florfenicol Amine
Residues in Poultry Meat and Pork via ASE-UPLC-FLD. J. Food Compos. Anal..

[64] Boitor R. A., Tódor I. S., Leopold L. F., Leopold N. (2015). Room Temperature
Synthesis
of Highly Monodisperse and Sers-Active Glucose-Reduced Gold Nanoparticles. J. Appl. Spectrosc..

[65] Kanda M., Kusano T., Kanai S., Hayashi H., Matushima Y., Nakajima T., Takeba K., Sasamoto T., Nagayma T. (2010). Rapid Determination
of Fluoroquinolone Residues in Honey by a Microbiological Screening
Method and Liquid Chromatography. J. AOAC Int..

[66] Duan N., Chang Y., Su T., Zhang X., Lu M., Wang Z., Wu S. (2024). Generation of a Specific Aptamer
for Accurate Detection of Sarafloxacin Based on Fluorescent/Colorimetric/SERS
Triple-Readout Sensor. Biosens. Bioelectron..

[67] Pumera M. (2013). Electrochemistry
of Graphene, Graphene Oxide and Other Graphenoids: Review. Electrochem. Commun..

[68] Sriram B., Baby J. N., Hsu Y.-F., Wang S.-F., George M. (2023). Scheelite-Type
Rare Earth Vanadates TVO4 (T = Ho, Y, Dy) Electrocatalysts: Investigation
and Comparison of T Site Variations towards Bifunctional Electrochemical
Sensing Application. Chem. Eng. J..

[69] Charoenraks T. (2004). Flow Injection
Analysis of Doxycycline or Chlortetracycline in Pharmaceutical Formulations
with Pulsed Amperometric Detection. Talanta.

[70] Bellan L. M., Craighead H. G. (2009). Nanomanufacturing Using Electrospinning. J. Manuf. Sci. Eng..

[71] Zhou J., Chen A., Guo H., Li Y., He X., Chen L., Zhang Y. (2022). Covalent Organic Framework/Polyacrylonitrile
Electrospun Nanofiber for Dispersive Solid-Phase Extraction of Trace
Quinolones in Food Samples. Nanomaterials.

[72] Liu M., Jia M., E Y., Li D. (2021). A Novel Ion Selective Electrode Based
on Reduced Graphene Oxide for Potentiometric Determination of Sarafloxacin
Hydrochloride. Microchem. J..

[73] Eldin G. M. G., Khalifa M. E., Munshi A. M., Aldawsari A. M., El-Metwaly N. M. (2021). Determining Nadifloxacin in Pharmaceutical Formulations
Using Novel Differential Pulse Voltammetric Approach. Microchem. J..

[74] Batra D., Shea K. J. (2003). Combinatorial Methods in Molecular Imprinting. Curr. Opin. Chem. Biol..

[75] Arnold, B. R. ; Euler, A. C. ; Jenkins, A. L. ; Uy, O. M. ; Murray, G. M. Progress in the Development of Molecularly Imprinted Polymer Sensors Johns hopkins univ laurel md applied physics lab 1999 20 2 191

[76] Wulff G., Sarhan A. (1972). Über Die Anwendung von Enzymanalog Gebauten
Polymeren Zur Racemattrennung. Angew. Chem.,
Int. Ed..

[77] Dickey F. H. (1955). Specific
Adsorption. J. Phys. Chem..

[78] Faysal A. A., Kaya S. I., Cetinkaya A., Ozkan S. A., Gölcü A. (2024). The Effect
of Polymerization Techniques on the Creation of Molecularly Imprinted
Polymer Sensors and Their Application on Pharmaceutical Compounds. Crit. Rev. Anal. Chem..

[79] Andersson L. I. (2000). Molecular
Imprinting: Developments and Applications in the Analytical Chemistry
Field. J. Chromatogr. B: biomed. Sci. Appl..

[80] Yi L.-X., Fang R., Chen G.-H. (2013). Molecularly Imprinted Solid-Phase
Extraction in the Analysis of Agrochemicals. J. Chromatogr. Sci..

[81] Cormack P. A. G., Elorza A. Z. (2004). Molecularly Imprinted Polymers: Synthesis and Characterisation. J. Chromatogr. B: Biomed. Sci. Appl..

[82] Elugoke S. E., Adekunle A. S., Fayemi O. E., Akpan E. D., Mamba B. B., Sherif E. M., Ebenso E. E. (2021). Molecularly
Imprinted Polymers (MIPs)
Based Electrochemical Sensors for the Determination of Catecholamine
Neurotransmitters – Review. Electrochem.
Sci. Adv..

[83] Qu J. R., Zhang J. J., Gao Y. F., Yang H. (2012). Synthesis and Utilisation
of Molecular Imprinting Polymer for Clean-up of Propachlor in Food
and Environmental Media. Food Chem..

[84] Komiyama, M. ; Takeuchi, T. ; Mukawa, T. ; Asanuma, H. Molecular Imprinting; Wiley,2002. 10.1002/352760202X

[85] Chapuis F., Pichon V., Hennion M.-C. (2004). Molecularly
Imprinted Polymers: Developments
and Applications of New Selective Solid-Phase Extraction Materials. lc-Gc Europe.

[86] Yan H., Row K. H. (2006). Characteristic and
Synthetic Approach of Molecularly
Imprinted Polymer. Int. J. Mol. Sci..

[87] Molecularly Imprinted Polymers: Man-Made Mimics of Antibodies and Their Application in Analytical Chemistry, Sellergren, B. , Eds.; Elsevier, 2000.

[88] da
Silva P. H. R., Diniz M. L. V., Pianetti G. A., da Costa
César I., Ribeiro e Silva M. E.
S., de Souza
Freitas R. F., de Sousa R. G., Fernandes C. (2018). Molecularly
Imprinted Polymer for Determination of Lumefantrine in Human Plasma
through Chemometric-Assisted Solid-Phase Extraction and Liquid Chromatography. Talanta.

[89] Pourfarzib M., Shekarchi M., Rastegar H., Akbari-Adergani B., Mehramizi A., Dinarvand R. (2015). Molecularly Imprinted Nanoparticles
Prepared by Miniemulsion Polymerization as a Sorbent for Selective
Extraction and Purification of Efavirenz from Human Serum and Urine. J. Chromatogr. B: Biomed. Sci. Appl..

[90] Tawab M. A. H. A., El-Moghny M. G. A., El Nashar R. M. (2020). Computational
Design of Molecularly Imprinted Polymer for Electrochemical Sensing
and Stability Indicating Study of Sofosbuvir. Microchem. J..

[91] Güney S. (2023). An Electrochemical
Sensor Based on Molecularly Imprinted Polydopamine Coated on Reduced
Graphene Oxide for Selective Detection of Ornidazole. Electroanalysis.

[92] Manzoor S., Buffon R., Rossi A. V. (2015). Molecularly Imprinted Solid Phase
Extraction of Fluconazole from Pharmaceutical Formulations. Talanta.

[93] Vasapollo G., Sole R. D., Mergola L., Lazzoi M. R., Scardino A., Scorrano S., Mele G. (2011). Molecularly Imprinted
Polymers: Present
and Future Prospective. Int. J. Mol. Sci..

[94] Tamayo F. G., Turiel E., Martín-Esteban A. (2007). Molecularly Imprinted
Polymers for Solid-Phase Extraction and Solid-Phase Microextraction:
Recent Developments and Future Trends. J. Chromatogr.
A.

[95] Sellergren B. (1994). Direct Drug
Determination by Selective Sample Enrichment on an Imprinted Polymer. Anal. Chem..

[96] Caro E., Marce R., Cormack P., Sherrington D., Borrull F. (2004). A New Molecularly Imprinted Polymer
for the Selective
Extraction of Naproxen from Urine Samples by Solid-Phase Extraction. J. Chromatogr. B: Biomed. Sci. Appl..

[97] Hu X., Hu Y., Li G. (2007). Development
of Novel Molecularly Imprinted Solid-Phase
Microextraction Fiber and Its Application for the Determination of
Triazines in Complicated Samples Coupled with High-Performance Liquid
Chromatography. J. Chromatogr. A.

[98] Zhu X., Zhu Q. (2008). Molecular Imprinted
Nylon-6 Stir Bar as a Novel Extraction Technique
for Enantioseparation of Amino Acids. J. Appl.
Polym. Sci..

[99] Kröger S., Turner A. P. F., Mosbach K., Haupt K. (1999). Imprinted Polymer-Based
Sensor System for Herbicides Using Differential-Pulse Voltammetry
on Screen-Printed Electrodes. Anal. Chem..

[100] Chi H., Liu G. (2023). Carbon Nanomaterial-Based
Molecularly Imprinted Polymer
Sensors for Detection of Hazardous Substances in Food: Recent Progress
and Future Trends. Food Chem..

[101] Meng F., Duan M., Wu W., Shao S., Qin Y., Zhang M. (2024). Enzymatic Construction
Au NPs-RGO Based MIP Electrochemical
Sensor for Adulteration Detection of Bovine-Derived Allergen in Camel
Milk. Food Chem..

[102] Apak R., Üzer A., SaĞlam Ş., Arman A. (2023). Selective Electrochemical Detection
of Explosives with
Nanomaterial Based Electrodes. Electroanalysis.

[103] Mahmoud A. M., El-Wekil M. M., Mahnashi M. H., Ali M. F. B., Alkahtani S. A. (2019). Modification of N, S Co-Doped Graphene
Quantum Dots
with p-Aminothiophenol-Functionalized Gold Nanoparticles for Molecular
Imprint-Based Voltammetric Determination of the Antiviral Drug Sofosbuvir. Microchim. Acta.

[104] El-Wekil M. M., Hayallah A. M., Abdelgawad M. A., Abourehab M. A. S., Shahin R. Y. (2022). Nanocomposite of Gold Nanoparticles@nickel
Disulfide-Plant Derived Carbon for Molecularly Imprinted Electrochemical
Determination of Favipiravir. J. Electroanal.
Chem..

[105] Cetinkaya A., Unal M. A., Nazır H., Çorman M. E., Uzun L., Ozkan S. A. (2024). Development of Borazine-Assisted-Oriented
Molecularly Imprinted Electrochemical Sensor for the Detection of
Umifenovir in Serum and Urine by EIS and DPV Methods. Sens. Actuators, B.

[106] Erk N., Mehmandoust M., Soylak M. (2022). Electrochemical Sensing of Favipiravir
with an Innovative Water-Dispersible Molecularly Imprinted Polymer
Based on the Bimetallic Metal-Organic Framework: Comparison of Morphological
Effects. Biosensors.

[107] Parvinizadeh F., Daneshfar A. (2019). Fabrication
of a Magnetic Metal–Organic
Framework Molecularly Imprinted Polymer for Extraction of Anti-Malaria
Agent Hydroxychloroquine. New J. Chem..

[108] Wang P., Ge L., Li M., Li W., Li L., Wang Y., Yu J. (2013). Photoelectrochemical Sensor Based
on Molecularly Imprinted Polymer-Coated TiO2 Nanotubes for Lindane
Specific Recognition and Detection. J. Inorg.
Organomet. Polym. Mater..

[109] Zhang Z., Zhou H., Jiang C., Wang Y. (2020). Molecularly
Imprinted Polymer Functionalized Flower-like BiOBr Microspheres for
Photoelectrochemical Sensing of Chloramphenicol. Electrochim. Acta.

[110] Potyrailo R. A. (2006). Polymeric
Sensor Materials: Toward an Alliance of Combinatorial
and Rational Design Tools?. Angew. Chem., Int.
Ed..

[111] Dickert F. L., Forth F. P. G. J., Lieberzeit P., Tortschanoff M. (1998). Molecular
Imprinting in Chemical Sensing – Detection
of Aromatic and Halogenated Hydrocarbons as Well as Polar Solvent
Vapors. Fresenius’ J. Anal. Chem..

[112] Kepley L. J., Crooks’ R. M., Ricco’ A. J. (1992). A selective
SAW-based organophosphonate chemical sensor employing a self-assembled,
composite monolayer: a new paradigm for sensor design. Anal. Chem..

[113] Pan Y., Yang L., Mu N., Shao S., Wang W., Xie X., He S. (2014). A SAW-Based
Chemical Sensor for Detecting Sulfur-Containing
Organophosphorus Compounds Using a Two-Step Self-Assembly and Molecular
Imprinting Technology. Sensors.

[114] Yan S., Fang Y., Gao Z. (2007). Quartz Crystal Microbalance for the
Determination of Daminozide Using Molecularly Imprinted Polymers as
Recognition Element. Biosens. Bioelectron..

[115] Rajkumar R., Katterle M., Warsinke A., Möhwald H., Scheller F. W. (2008). Thermometric MIP Sensor for Fructosyl Valine. Biosens. Bioelectron..

[116] Vulic K., Shoichet M. S. (2014). Affinity-Based Drug
Delivery Systems
for Tissue Repair and Regeneration. Biomacromolecules.

[117] Stanley S., Percival C. J., Morel T., Braithwaite A., Newton M. I., McHale G., Hayes W. (2003). Enantioselective
Detection
of L-Serine. Sens. Actuators, B.

[118] Iacob B. C., Bodoki E., Farcau C., Barbu-Tudoran L., Oprean R. (2016). Study of the Molecular Recognition Mechanism of an
Ultrathin MIP Film-Based Chiral Electrochemical Sensor. Electrochim. Acta.

[119] Iacob B. C., Bodoki E., Florea A., Bodoki A. E., Oprean R. (2015). Simultaneous Enantiospecific Recognition
of Several
β-Blocker Enantiomers Using Molecularly Imprinted Polymer-Based
Electrochemical Sensor. Anal. Chem..

[120] Alharbi H.
Y., Alnoman R. B., Aljohani M. S., Monier M. (2025). Development
of Clickable Imprinted Polymer for Enantioselective Recognition and
Separation of S-Propranolol Enantiomer. Microchem.
J..

[121] Lagarde F. (2013). MIP-Based Impedimetric Sensors. Key Eng. Mater..

[122] Küçük D., Üner G., İpek S. L., Caglayan M. O., ÜstündaĞ Z. (2024). An Impedimetric
Determination of Zearalenone on MIP-Modified Carboceramic Electrode. Toxicon.

[123] Arshad R., Rhouati A., Hayat A., Nawaz M. H., Yameen M. A., Mujahid A., Latif U. (2020). MIP-Based Impedimetric
Sensor for Detecting Dengue Fever Biomarker. Appl. Biochem. Biotechnol..

[124] Mazouz Z., Rahali S., Fourati N., Zerrouki C., Aloui N., Seydou M., Yaakoubi N., Chehimi M., Othmane A., Kalfat R. (2017). Highly Selective Polypyrrole
MIP-Based
Gravimetric and Electrochemical Sensors for Picomolar Detection of
Glyphosate. Sensors.

[125] Zhou Y., He J., Wang H., Nan N., Qi K., Cui S. (2017). Fabrication of Superhydrophobic Nanofiber
Fabric with
Hierarchical Nanofiber Structure. E-Polymers.

[126] Anirudhan T. S., Alexander S. (2015). Design and Fabrication of Molecularly
Imprinted Polymer-Based Potentiometric Sensor from the Surface Modified
Multiwalled Carbon Nanotube for the Determination of Lindane (γ-Hexachlorocyclohexane),
an Organochlorine Pesticide. Biosens. Bioelectron..

[127] Heravizadeh O. R., Khadem M., Nabizadeh R., Shahtaheri S. J. (2019). Synthesis of Molecularly Imprinted Nanoparticles for
Selective Exposure Assessment of Permethrin: Optimization by Response
Surface Methodology. J. Environ. Health Sci.
Eng..

[128] Vonderheide A. P., Boyd B., Ryberg A., Yilmaz E., Hieber T. E., Kauffman P. E., Garris S. T., Morgan J. N. (2009). Analysis
of Permethrin Isomers in Composite Diet Samples by Molecularly Imprinted
Solid-Phase Extraction and Isotope Dilution Gas Chromatography–Ion
Trap Mass Spectrometry. J. Chromatogr. A.

[129] Ma G., Chen L. (2014). Development
of Magnetic Molecularly Imprinted Polymers
Based on Carbon Nanotubes – Application for Trace Analysis
of Pyrethroids in Fruit Matrices. J. Chromatogr.
A.

[130] Zhang M., He J., Shen Y., He W., Li Y., Zhao D., Zhang S. (2018). Application of Pseudo-Template
Molecularly
Imprinted Polymers by Atom Transfer Radical Polymerization to the
Solid-Phase Extraction of Pyrethroids. Talanta.

[131] Wang H., Qian D., Xiao X., He B., Gao S., Shi H., Liao L., Deng J. (2017). Enantioselective
Determination
of S-Ornidazole by Using Carbon Paste Electrode Modified with Boron-Embedded
Conductive Copolymer-Polysiloxane-Based Molecularly Imprinted Hybrid
Film. Electrochim. Acta.

[132] Mehrzad-Samarin M., Faridbod F., Ganjali M. R. (2019). A Luminescence
Nanosensor
for Ornidazole Detection Using Graphene Quantum Dots Entrapped in
Silica Molecular Imprinted Polymer. Spectrochim.
Acta, Part A.

[133] Liao S., Weng Q. (2018). Rapid Separation and Determination
of Metronidazole Benzoate and Other Antiprotozoal Drugs by Pressurized
Capillary Electrochromatography. J. Chem..

[134] Pan Y., Liu X., Liu J., Wang J., Liu J., Gao Y., Ma N. (2022). Chemiluminescence
Sensors Based on Molecularly Imprinted
Polymers for the Determination of Organophosphorus in Milk. J. Dairy Sci..

[135] Liu W., Guo Y., Luo J., Kou J., Zheng H., Li B., Zhang Z. (2015). A Molecularly Imprinted
Polymer Based a Lab-on-Paper
Chemiluminescence Device for the Detection of Dichlorvos. Spectrochim. Acta, Part A.

[136] Xu Z., Fang G., Wang S. (2010). Molecularly
Imprinted Solid Phase
Extraction Coupled to High-Performance Liquid Chromatography for Determination
of Trace Dichlorvos Residues in Vegetables. Food Chem..

[137] Huang S., Tan L., Zhang L., Wu J., Zhang L., Tang Y., Wang H., Liang Y. (2020). Molecularly
Imprinted Mesoporous Silica Embedded with Perovskite CsPbBr3 Quantum
Dots for the Fluorescence Sensing of 2,2-Dichlorovinyl Dimethyl Phosphate. Sens. Actuators, B.

[138] Chen J., Zhang W., Shu Y., Ma X., Cao X. (2017). Detection
of Organophosphorus Pesticide Residues in Leaf Lettuce
and Cucumber Through Molecularly Imprinted Solid-Phase Extraction
Coupled to Gas Chromatography. Food Anal. Methods.

[139] Xin J., Qiao X., Ma Y., Xu Z. (2012). Simultaneous Separation
and Determination of Eight Organophosphorous Pesticide Residues in
Vegetables through Molecularly Imprinted Solid-Phase Extraction Coupled
to Gas Chromatography. J. Sep. Sci..

[140] Radi A.-E., Abd-Elaziz I. (2015). A Halofuginone Electrochemical Sensor
Based on a Molecularly Imprinted Polypyrrole Coated Glassy Carbon
Electrode. Anal. Methods.

[141] Peng H. (2000). Bulk Acoustic Wave Sensor Using Molecularly
Imprinted Polymers as
Recognition Elements for the Determination of Pyrimethamine. Talanta.

[142] Huang S., Guo M., Tan L., Tan J., Wu J., Tang Y., Liang Y. (2018). Click Chemistry-Based Core–Shell
Molecularly Imprinted Polymers for the Determination of Pyrimethamine
in Fish and Plasma Samples. Anal. Methods.

[143] Bidaud A.-L., Schwarz P., Herbreteau G., Dannaoui E. (2021). Techniques for the
Assessment of In Vitro and In Vivo
Antifungal Combinations. J. Fungi.

[144] Sun X., Wang M., Yang L., Wen H., Wang L., Li T., Tang C., Yang J. (2019). Preparation
and Evaluation of Dummy-Template
Molecularly Imprinted Polymer as a Potential Sorbent for Solid Phase
Extraction of Imidazole Fungicides from River Water. J. Chromatogr. A.

[145] Zhang X., Sun X., Wang M., Wang Y., Wu Q., Ji L., Li Q., Yang J., Zhou Q. (2020). Dummy Molecularly
Imprinted Microspheres Prepared by Pickering Emulsion Polymerization
for Matrix Solid-Phase Dispersion Extraction of Three Azole Fungicides
from Fish Samples. J. Chromatogr. A.

[146] Yang Y.-H., Liu L.-T., Chen M.-J., Liu S., Gong C.-B., Wei Y.-B., Chow C.-F., Tang Q. (2018). A Photoresponsive
Surface Molecularly Imprinted Polymer Shell for Determination of Trace
Griseofulvin in Milk. Mater. Sci. Eng..

[147] Bashir K., Guo P., Chen G., Li Y., Ge Y., Shu H., Fu Q. S. (2020). Characterization,
and Application
of Griseofulvin Surface Molecularly Imprinted Polymers as the Selective
Solid Phase Extraction Sorbent in Rat Plasma Samples. Arabian J. Chem..

[148] Bashir K., Luo Z., Chen G., Shu H., Cui X., Li W., Lu W., Fu Q. (2020). Development
of Surface
Molecularly Imprinted Polymers as Dispersive Solid Phase Extraction
Coupled with HPLC Method for the Removal and Detection of Griseofulvin
in Surface Water. Int. J. Environ. Res. Public
Health.

[149] Santos da Silva R. C., Mano V., Pereira A. C., Costa
de Figueiredo E., Borges K. B. (2016). Development of Pipette Tip-Based
on Molecularly Imprinted Polymer Micro-Solid Phase Extraction for
Selective Enantioselective Determination of (−)-(2S,4R) and
(+)-(2R,4S) Ketoconazole in Human Urine Samples Prior to HPLC-DAD. Anal. Methods.

[150] Adamson C. S., Chibale K., Goss R. J. M., Jaspars M., Newman D. J., Dorrington R. A. (2021). Antiviral
Drug Discovery: Preparing
for the next Pandemic. Chem. Soc. Rev..

[151] Mtolo S. P., Mahlambi P. N., Madikizela L. M. (2019). Synthesis
and Application of a Molecularly Imprinted Polymer in Selective Solid-Phase
Extraction of Efavirenz from Water. Water Sci.
Technol..

[152] Xolo T., Mahlambi P. (2024). Molecularly Imprinted Polymers as
Solid-Phase and Dispersive Solid-Phase Extraction Sorbents in the
Extraction of Antiretroviral Drugs in Water: Adsorption, Selectivity
and Reusability Studies. J Anal Sci Technol..

[153] Simões N. S., de Oliveira H. L., da Silva R. C. S., Teixeira L. S., Sales T. L. S., de
Castro W. V., de Paiva M. J. N., Sanches C., Borges K. B. (2018). Hollow
Mesoporous Structured Molecularly Imprinted
Polymer as Adsorbent in Pipette-tip Solid-phase Extraction for the
Determination of Antiretrovirals from Plasma of HIV-infected Patients. Electrophoresis.

[154] Soliman M. A., Mahmoud A. M., Elzanfaly E. S., Abdel Fattah L. E. (2023). Electrochemical Sensor Based on Bio-Inspired Molecularly
Imprinted Polymer for Sofosbuvir Detection. RSC Adv..

[155] Mahmoud A. M., El-Wekil M. M., Mahnashi M. H., Ali M. F. B., Alkahtani S. A. (2019). Modification
of N,S Co-Doped Graphene Quantum Dots
with p-Aminothiophenol-Functionalized Gold Nanoparticles for Molecular
Imprint-Based Voltammetric Determination of the Antiviral Drug Sofosbuvir. Microchim. Acta.

[156] Yamani H. Z., El Azab N. F. (2024). First Electropolymerized Carbidopa-Based
Molecularly Imprinted Film: Disposable Electrochemical Sensor for
Monitoring of Anti-COVID-19 Drug Favipiravir in Human Plasma. Microchem. J..

[157] Wang S., Wang C., Xin Y., Li Q., Liu W. (2022). Core–Shell
Nanocomposite of Flower-like Molybdenum Disulfide
Nanospheres and Molecularly Imprinted Polymers for Electrochemical
Detection of Anti COVID-19 Drug Favipiravir in Biological Samples. Microchim. Acta.

[159] Mahdavi Nejad E. (2024). Simultaneous Electrochemical Quantification
of Favipiravir
and Molnupiravir as Antiviral Drugs for the Treatment of COVID-19
by Using a Glassy Carbon Electrode Modified by Pd/Co-Mn-MOF-74. J. Electrochem. Soc..

[160] Pauter K., Szultka-Młyńska M., Buszewski B. (2020). Determination and Identification of Antibiotic Drugs
and Bacterial Strains in Biological Samples. Molecules.

[161] Zhang Y., Zhang Y., Jamal R., Abdiryim T. (2024). Highly Sensitive
Electrochemical Sensing of Moxifloxacin Based on Molecularly Imprinted
and Au Nanoparticle Functionalized PEDOT Composites. Chem. Eng. J..

[162] Hammam M. A., Abdel-Halim M., Madbouly A., Wagdy H. A., El Nashar R. M. (2019). Computational
Design of Molecularly Imprinted Polymer
for Solid Phase Extraction of Moxifloxacin Hydrochloride from Avalox®
Tablets and Spiked Human Urine Samples. Microchem.
J..

[163] Hammam M. A., Wagdy H. A., El Nashar R. M. (2018). Moxifloxacin
Hydrochloride Electrochemical Detection Based on Newly Designed Molecularly
Imprinted Polymer. Sens. Actuators, B.

[164] Amjadi M., Jalili R., Manzoori J. L. (2016). A Sensitive
Fluorescent
Nanosensor for Chloramphenicol Based on Molecularly Imprinted Polymer-capped
CdTe Quantum Dots. Luminescence.

[165] Zhao F., She Y., Zhang C., Wang S., Du X., Jin F., Jin M., Shao H., Zheng L., Wang J. (2017). Selective Determination
of Chloramphenicol in Milk Samples by the
Solid-Phase Extraction Based on Dummy Molecularly Imprinted Polymer. Food Anal. Methods.

[166] Wu C., Cheng R., Wang J., Wang Y., Jing X., Chen R., Sun L., Yan Y. (2018). Fluorescent Molecularly
Imprinted Nanoparticles for Selective and Rapid Detection of Ciprofloxacin
in Aquaculture Water. J. Sep. Sci..

[167] Li S., Pang C., Ma X., Wu Y., Wang M., Xu Z., Luo J. (2022). Aggregation-Induced
Electrochemiluminescence and Molecularly
Imprinted Polymer Based Sensor with Fe3O4@Pt Nanoparticle Amplification
for Ultrasensitive Ciprofloxacin Detection. Microchem. J..

[168] Li Z., Cui Z., Tang Y., Liu X., Zhang X., Liu B., Wang X., Draz M. S., Gao X. (2019). Fluorometric Determination
of Ciprofloxacin Using Molecularly Imprinted Polymer and Polystyrene
Microparticles Doped with Europium­(III)­(DBM)­3phen. Microchim. Acta.

[169] Huang Y., Sun X., Yang J., Cao Z., Wang R., Li L., Ding Y. (2023). A Molecularly Imprinted
Electrochemical Sensor with Dual Functional Monomers for Selective
Determination of Gatifloxacin. Microchim. Acta.

[170] Zhao L., Liu G., Zheng B., Wang G., Wang Y., Liu L., Sun C., Ma X. (2023). Molecularly
Imprinted Two-dimensional Photonic Crystal Hydrogel Sensor for the
Detection of Gatifloxacin. Polym. Int..

[171] Chen J., Tan L., Cui Z., Qu K., Wang J. (2022). Graphene Oxide Molecularly Imprinted Polymers as Novel
Adsorbents
for Solid-Phase Microextraction for Selective Determination of Norfloxacin
in the Marine Environment. Polymers.

[172] Shi T., Fu H., Tan L., Wang J. (2019). CdTe Quantum Dots Coated
with a Molecularly Imprinted Polymer for Fluorometric Determination
of Norfloxacin in Seawater. Microchim. Acta.

[173] Zhang Z., Wu T., Zhou H., Jiang C., Wang Y. (2021). 3D Flower-Shaped BiOI Encapsulated
in Molecularly Imprinted Polymer
for Hypersensitivity to Norfloxacin. Microchem.
J..

[174] Zhang Z., Huang L., Sheng S., Jiang C., Wang Y. (2021). MIL-125­(Ti)-Derived COOH Functionalized
TiO2 Grafted Molecularly
Imprinted Polymers for Photoelectrochemical Sensing of Ofloxacin. Sens. Actuators, B.

[175] Wang Y.-Q., Fang Z., Min H., Xu X.-Y., Li Y. (2022). Sensitive
Determination of Ofloxacin by Molecularly Imprinted Polymers
Containing Ionic Liquid Functionalized Carbon Quantum Dots and Europium
Ion. ACS Appl. Nano Mater..

[176] Qi Y., Chen Y., Li Q., Dang X., Chen H. (2024). A Novel Ratiometric
Electrochemical Sensing Platform Combined with Molecularly Imprinted
Polymer and Fe-MOF-NH2/CNTs-NH2/MXene Composite for Efficient Detection
of Ofloxacin. Anal. Chim. Acta.

[177] Zhang Z., Hu Y., Zhang H., Yao S. (2010). Novel Layer-by-Layer
Assembly Molecularly Imprinted Sol–Gel Sensor for Selective
Recognition of Clindamycin Based on Au Electrode Decorated by Multi-Wall
Carbon Nanotube. J. Colloid Interface Sci..

[178] Fan Y., Zeng G., Ma X. (2020). Multi-Templates Surface Molecularly
Imprinted Polymer for Rapid Separation and Analysis of Quinolones
in Water. Environ. Sci. Pollut. Res..

[179] Tang Y., Liu H., Gao J., Liu X., Gao X., Lu X., Fang G., Wang J., Li J. (2018). Upconversion
Particle@Fe3O4@molecularly Imprinted Polymer with Controllable Shell
Thickness as High-Performance Fluorescent Probe for Sensing Quinolones. Talanta.

[180] Urraca J. L., Chamorro-Mendiluce R., Orellana G., Moreno-Bondi M. C. (2016). Molecularly
Imprinted Polymer Beads for Clean-up and Preconcentration of β-Lactamase-Resistant
Penicillins in Milk. Anal. Bioanal. Chem..

[181] Shi X., Zuo Y., Jia X., Wu X., Jing N., Wen B., Mi X. (2020). A Novel Molecularly Imprinted Sensor Based on Gold
Nanoparticles/Reduced Graphene Oxide/Single-Walled Carbon Nanotubes
Nanocomposite for the Detection of Pefloxacin. Int. J. Electrochem. Sci..

[182] Li G., Qi X., Wu J., Wan X., Wang T., Liu Y., Chen Y., Xia Y. (2024). Highly Stable
Electrochemical Sensing
Platform for the Selective Determination of Pefloxacin in Food Samples
Based on a Molecularly Imprinted-Polymer-Coated Gold Nanoparticle/Black
Phosphorus Nanocomposite. Food Chem..

[183] Liu X., Pu J., Li J., Gong B. (2022). Preparation and Performance
Analysis of Monodisperse Glycidyl Methacrylate Modified Restricted
Access Media-imprinted Materials. J. Sep. Sci..

[184] Tan F., Zhai M., Meng X., Wang Y., Zhao H., Wang X. (2021). Hybrid Peptide-Molecularly Imprinted Polymer Interface for Electrochemical
Detection of Vancomycin in Complex Matrices. Biosens. Bioelectron..

[185] Zhou H., Chen Q., Song X., He L., Liu R. (2022). Surface Molecularly
Imprinted Solid-Phase Extraction for the Determination
of Vancomycin in Plasma Samples Using HPLC–MS/MS. Anal. Sci..

[186] Cetinkaya A., Yıldız E., Kaya S. I., Çorman M. E., Uzun L., Ozkan S. A. (2022). A Green
Synthesis Route to Develop
Molecularly Imprinted Electrochemical Sensor for Selective Detection
of Vancomycin from Aqueous and Serum Samples. Green Anal. Chem..

[187] Szultka M., Krzeminski R., Jackowski M., Buszewski B. (2013). Simultaneous
Determination of Selected Chemotherapeutics
in Human Whole Blood by Molecularly Imprinted Polymers Coated Solid
Phase Microextraction Fibers and Liquid Chromatography–Tandem
Mass Spectrometry. J. Chromatogr. B: Biomed.
Sci. Appl..

[188] Peng J., Liu D., Shi T., Tian H., Hui X., He H. (2017). Molecularly
Imprinted Polymers Based Stir Bar Sorptive
Extraction for Determination of Cefaclor and Cefalexin in Environmental
Water. Anal. Bioanal. Chem..

[189] Orachorn N., Bunkoed O. (2019). A Nanocomposite Fluorescent
Probe
of Polyaniline, Graphene Oxide and Quantum Dots Incorporated into
Highly Selective Polymer for Lomefloxacin Detection. Talanta.

[190] Li J., Huang X., Ma J., Wei S., Zhang H. (2020). A Novel Electrochemical
Sensor Based on Molecularly Imprinted Polymer with Binary Functional
Monomers at Fe-Doped Porous Carbon Decorated Au Electrode for the
Sensitive Detection of Lomefloxacin. Ionics.

[191] Zhu G., Li W., Wang P., Cheng G., Chen L., Zhang K., Li X. (2020). One-step Polymerization of Hydrophilic
Ionic Liquid Imprinted Polymer in Water for Selective Separation and
Detection of Levofloxacin from Environmental Matrices. J. Sep. Sci..

[192] Zheng B., Liu G., Zhao L., Wang G., Wang Y. (2022). Levofloxacin Molecularly Imprinted Two Dimensional Photonic Crystal
Hydrogel Sensor. Polymer.

[193] El Azab N. F., Mahmoud A. M., Trabik Y. A. (2022). Point-of-Care Diagnostics
for Therapeutic Monitoring of Levofloxacin in Human Plasma Utilizing
Electrochemical Sensor Mussel-Inspired Molecularly Imprinted Copolymer. J. Electroanal. Chem..

[194] Garcinuño R. M., Collado E. J., Paniagua G., Bravo J. C., Fernández
Hernando P. (2023). Assessment of Molecularly Imprinted
Polymers as Selective Solid-Phase Extraction Sorbents for the Detection
of Cloxacillin in Drinking and River Water. Polymers.

[195] Jafari S., Dehghani M., Nasirizadeh N., Baghersad M. H., Azimzadeh M. (2019). Label-Free Electrochemical Detection
of Cloxacillin Antibiotic in Milk Samples Based on Molecularly Imprinted
Polymer and Graphene Oxide-Gold Nanocomposite. Measurement.

[196] Du W., Sun M., Guo P., Chang C., Fu Q. (2018). Molecularly
Imprinted Membrane Extraction Combined with High-Performance Liquid
Chromatography for Selective Analysis of Cloxacillin from Shrimp Samples. Food Chem..

[197] Abdallah N. A., Ibhrahim H. F., Hegabe N. H. (2017). Comparative
Study
of Molecularly Imprinted Polymer and Magnetic Molecular Imprinted
Nanoparticles as Recognition Sites for the Potentiometric Determination
of Gemifloxacin Mesylate. Int. J. Electrochem.
Sci..

[198] Omran N. H., Wagdy H. A., Abdel-Halim M., Nashar R. M. E. (2019). Validation and Application of Molecularly Imprinted
Polymers for SPE/UPLC–MS/MS Detection of Gemifloxacin Mesylate. Chromatographia.

[199] Geng Y., Guo M., Tan J., Huang S., Tang Y., Tan L., Liang Y. (2019). The Fabrication
of
Highly Ordered Fluorescent Molecularly Imprinted Mesoporous Microspheres
for the Selective Sensing of Sparfloxacin in Biological Samples. Sens. Actuators, B.

[200] Chansud N., Kaewnok R., Nurerk P., Davis F., Bunkoed O. (2023). Ultrasensitive and Highly Selective Fluorescence Probe
of Nitrogen-Doped Graphene Quantum Dots and Zinc Oxide Decorated Carbon
Foam Incorporated Molecularly Imprinted Polymer for Trace Sparfloxacin
Determination. Mater. Today Commun..

[201] Sa-Nguanprang S., Phuruangrat A., Bunkoed O. (2023). Fluorescent Probe of
Quantum Dots and Zinc Oxide in a Highly Selective Polymer Simultaneously
Determined Florfenicol and Sparfloxacin. Microchim.
Acta.

[202] Tan L., Li Y., Pan X., Marina M. L., Jiang Z. (2020). Boronate Affinity
Glycosyl Molecularly Imprinted Polymer Microspheres for the Determination
of Teicoplanin Using Ultra-High Performance Liquid Chromatography
Coupled with Tandem Mass Spectrometry. J. Chromatogr.
A.

[203] Zhou H., Peng K., Su Y., Song X., Qiu J., Xiong R., He L. (2021). Preparation
of Surface Molecularly
Imprinted Polymer and Its Application for the Selective Extraction
of Teicoplanin from Water. RSC Adv..

[204] Zhang Y., Li D., Tian X. (2024). A Highly Efficient
Fluorescent Turn-Off Nanosensor for Quantitative Detection of Teicoplanin
Antibiotic from Humans, Food, and Water Based on the Electron Transfer
between Imprinted Quantum Dots and the Five-Membered Cyclic Boronate
Esters. Molecules.

[205] Jin S., Zhang Y., Liu R., Liu Z., Gong L., Zhang L., Zhao T., Chen S., Fa H., Niu L., Yin W. (2025). A Novel Molecularly Imprinted Electrochemical
Sensor
Based on MnO-Fe3O4@C Was Designed with DFT Theoretical Study for the
Detection of Thiamphenicol in Food. Colloids
Surf., A.

[206] Chen S., Ouyang W., Zhu Y., Han J., Zhang Y., Zou L., Deng J., Liu A., Liu S., Yang Y. (2023). Development of a Green Simple Molecularly Imprinted
Nanoprobe for Rapid Determination of Trace Thiamphenicol Residual
in Animal Derived Foods. LWT.

[207] Yang G., Chen X., Pan Q., Liu W., Zhao F. (2017). A Novel Photoelectrochemical Sensor for Thiamphenicol Based on Porous
Three-Dimensional Imprinted Film. Int. J. Electrochem.
Sci..

[208] Ren Y., Fan Z. (2023). Synthesis of Molecularly Imprinted Polymers Based on
Nitrogen-Doped Carbon Dots for Specific Detection of Chlortetracycline
by Reversed Phase Microemulsion Method. Talanta.

[209] Zhang X., Li T., Gao X., Lin J., Zeng B., Gong C., Zhao F. (2023). Porphyrin and Molecularly
Imprinted Polymer Double Sensitized Cathode Photoelectrochemical Sensor
for Chlortetracycline. Microchem. J..

[210] Deng L., Liu J., Huang H., Deng C., Lu L., Wang L., Wang X. (2023). A Molecularly
Imprinted Electrochemical
Sensor Based on TiO2@Ti3C2Tx for Highly Sensitive and Selective Detection
of Chlortetracycline. Molecules.

[211] Li W., Sun C., Wang H., Bai Q., Xu Y., Bo C., Ou J. (2025). Detection and Adsorption
of Florfenicol in Milk Using
Bifunctional Carbon Dot–Doped Molecularly Imprinted Polymers. Electrophoresis.

[212] Guo S., Wu S., Zhao S., Wang X., Cai T., Li J., Gong B. (2021). Selective Removal of Florfenicol from Fetal Bovine
Serum by Restricted Access Media–Magnetic Molecularly Imprinted
Polymers. Chromatographia.

[213] Cao Y., Lu K., Chen Y., Zheng Q., Huang C., Jia N. (2023). In _2_ O _3_/Bi _2_ S _3_ S-Scheme
Heterojunction-Driven Molecularly Imprinted Photoelectrochemical Sensor
for Ultrasensitive Detection of Florfenicol. ACS Appl. Mater. Interfaces.

[214] Pongprom A., Chansud N., Sa-Nguanprang S., Jullakan S., Bunkoed O. (2023). Magnetic Fluorescent Probe of Hydroxylated-Halloysite
and Nitrogen-Doped Graphene Quantum Dots in Molecularly Imprinted
Polymer to Enrich and Determine Marbofloxacin. Microchem. J..

[215] Zhou L., Ye G., Yuan R., Chai Y., Chen S. (2007). A Capacitive Sensor
Based on Molecularly Imprinted Polymers and Poly­(p-Aminobenzene
Sulfonic Acid) Film for Detection of Pazufloxacin Mesilate. Sci. China B Chem..

[216] Tarek M., Elzanfaly E. S., Amer S. M., Wagdy H. A. (2020). Selective
Analysis of Nadifloxacin in Human Plasma Samples Using a Molecularly
Imprinted Polymer-Based Solid-Phase Extraction Proceeded by UPLC-DAD
Analysis. Microchem. J..

[217] Cai T., Zhou Y., Liu H., Li J., Wang X., Zhao S., Gong B. (2021). Preparation of Monodisperse,
Restricted-Access,
Media-Molecularly Imprinted Polymers Using Bi-Functional Monomers
for Solid-Phase Extraction of Sarafloxacin from Complex Samples. J. Chromatogr. A.

[218] Chaowana R., Bunkoed O. (2019). A Nanocomposite Probe of Polydopamine/Molecularly
Imprinted Polymer/Quantum Dots for Trace Sarafloxacin Detection in
Chicken Meat. Anal. Bioanal. Chem..

[219] Sun X., He J., Cai G., Lin A., Zheng W., Liu X., Chen L., He X., Zhang Y. (2010). Room Temperature Ionic
Liquid-mediated Molecularly Imprinted Polymer Monolith for the Selective
Recognition of Quinolones in Pork Samples. J.
Sep. Sci..

[220] Zheng M.-M., Gong R., Zhao X., Feng Y.-Q. (2010). Selective
Sample Pretreatment by Molecularly Imprinted Polymer Monolith for
the Analysis of Fluoroquinolones from Milk Samples. J. Chromatogr. A.

[221] Liu W., Wang J., Yu W., Wang X. (2020). Study on a Biomimetic
Enzyme-Linked Immunosorbent Assay for Rapid Detection of Flumequine
in Animal Foods. Food Anal. Methods.

[222] Li D., Wen Z., Lin J., Zeng J., Li Z., Ke F., Gao D., Wang D. (2023). Molecularly Imprinted Polymer Based
on Magnetic Porous Cellulose for Specific Adsorption of Tetracyclines:
Preparation, Characterization, Property Evaluation, and Application. Mater. Today Chem..

[223] Chen J., Tan L., Qu K., Cui Z., Wang J. (2022). Novel Electrochemical
Sensor Modified with Molecularly Imprinted
Polymers for Determination of Enrofloxacin in Marine Environment. Microchim. Acta.

[224] Gallegos-Tabanico A., Jimenez-Canale J., Hernandez-Leon S. G., Burgara-Estrella A. J., Encinas-Encinas J. C., Sarabia-Sainz J. A. (2022). Development
of an Electrochemical Sensor Conjugated with Molecularly Imprinted
Polymers for the Detection of Enrofloxacin. Chemosensors.

[225] Wang D., Jiang S., Liang Y., Wang X., Zhuang X., Tian C., Luan F., Chen L. (2022). Selective
Detection of Enrofloxacin in Biological and Environmental Samples
Using a Molecularly Imprinted Electrochemiluminescence Sensor Based
on Functionalized Copper Nanoclusters. Talanta.

[226] Wu S., Mao J., Zhang Y., Wang S., Huo M., Guo H. (2023). Sensitive Electrochemical
Detection of Enrofloxacin in Eggs Based
on Carboxylated Multi-Walled Carbon Nanotubes-Reduced Graphene Oxide
Nanocomposites: Molecularly Imprinted Recognition versus Direct Electrocatalytic
Oxidation. Food Chem..

[227] Hasanah A. N., Susanti I. (2023). Molecularly Imprinted
Polymers for
Pharmaceutical Impurities: Design and Synthesis Methods. Polymers.

